# An Endosomal Escape Trojan Horse Platform to Improve Cytosolic Delivery of Nucleic Acids

**DOI:** 10.1021/acsnano.3c09027

**Published:** 2024-02-12

**Authors:** Steven Narum, Brendan Deal, Hiroaki Ogasawara, Joseph Nicholas Mancuso, Jiahui Zhang, Khalid Salaita

**Affiliations:** † Department of Biomedical Engineering, Georgia Institute of Technology and Emory University, Atlanta, Georgia 30322, United States; ‡ Department of Chemistry, Emory University, Atlanta, Georgia 30322, United States

**Keywords:** i-Motif, endosomal escape, nucleic acid delivery, antisense therapy, nanomedicine, HIF1a

## Abstract

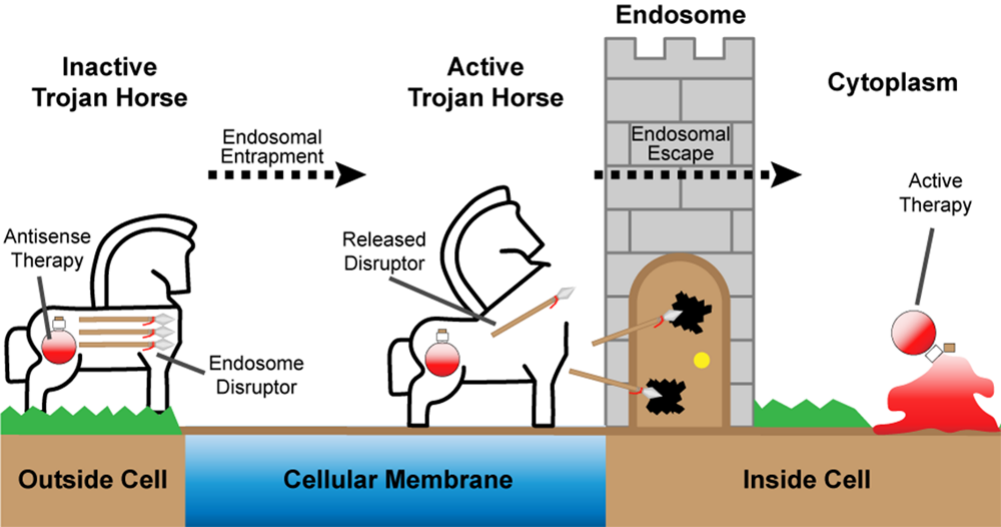

Endocytosis is a major bottleneck toward cytosolic delivery of nucleic acids, as the vast majority of nucleic acid drugs remain trapped within endosomes. Current trends to overcome endosomal entrapment and subsequent degradation provide varied success; however, active delivery agents such as cell-penetrating peptides have emerged as a prominent strategy to improve cytosolic delivery. Yet, these membrane-active agents have poor selectivity for endosomal membranes, leading to toxicity. A hallmark of endosomes is their acidic environment, which aids in degradation of foreign materials. Here, we develop a pH-triggered spherical nucleic acid that provides smart antisense oligonucleotide (ASO) release upon endosomal acidification and selective membrane disruption, termed DNA EndosomaL Escape Vehicle Response (DELVR). We anchor i-Motif DNA to a nanoparticle (AuNP), where the complement strand contains both an ASO sequence and a functionalized endosomal escape peptide (EEP). By orienting the EEP toward the AuNP core, the EEP is inactive until it is released through acidification-induced i-Motif folding. In this study, we characterize a small library of i-Motif duplexes to develop a structure-switching nucleic acid sequence triggered by endosomal acidification. We evaluate antisense efficacy using HIF1a, a hypoxic indicator upregulated in many cancers, and demonstrate dose-dependent activity through RT-qPCR. We show that DELVR significantly improves ASO efficacy *in vitro*. Finally, we use fluorescence lifetime imaging and activity measurement to show that DELVR benefits synergistically from nuclease- and pH-driven release strategies with increased ASO endosomal escape efficiency. Overall, this study develops a modular platform that improves the cytosolic delivery of nucleic acid therapeutics and offers key insights for overcoming intracellular barriers.

Antisense oligonucleotides (ASOs) are promising drugs to treat a broad range of diseases. ASOs are short oligonucleotides (16–20 bases long) designed to bind complementary mRNA and block translation.^1^ Mechanistically, ASOs can inhibit protein expression by recruiting RNase H to degrade the mRNA and also by steric blocking of the ribosome.^2^ For the past three decades, ASOs have been envisioned as a powerful class of drugs, but problems with the stability of oligonucleotides *in vivo* hindered their application. This changed with the advent of “third-generation” oligonucleotides with nuclease-resistant chemical modifications.^3^ Indeed, more ASOs have been FDA approved in the past five years than any other time in history, and dozens of new ASOs are being tested in the clinical trial pipeline.^4^ A second challenge that has hindered ASO drugs is the highly negatively charged backbone of the polymer and its large molecular weight. As predicted by Lipinski’s rule of five, these structural properties contribute to ASO’s failure to spontaneously cross the cell membrane to reach target mRNA. Unsurprisingly, it is estimated that less than ∼0.1% of nucleic acids make it into the cytoplasm of cells, and the vast majority of DNA drugs are trapped in endosomes.^3,5−7^ Within endosomes, the environment acidifies and eventually merges with lysosomes that contain degradative enzymes, destroying the cargo.^8,9^ The general workaround to the delivery issue is to dose ASOs at high concentrations, but large dosing causes off-target cytotoxicity and increased immune response while also increasing the overall cost of the therapy.^10^ Thrombocytopenia is the most common adverse event that has led to halting multiple ASO trials.^10^ Indeed, out of the 20,000 FDA approved drugs, only 16 are comprised of nucleic acids.^11^ As such, enhancing the efficacy of DNA drugs, even marginally, is highly desirable and may help transform this class of drugs.

Nanoparticles improve the pharmacokinetics of nucleic acid drugs, and specifically nanoparticles that minimize nonspecific immune response while delivering DNA cargo at therapeutic concentrations are actively being investigated and clinically tested.^1,10,12^ Broadly, these include vehicles such as lipids, liposomes, spherical nucleic acids (SNAs), and polymeric nanoparticles.^13,14^ A major benefit of using nanoparticles for nucleic acid delivery is that nucleic acids become less susceptible to enzymatic degradation, potentially through steric blocking of nucleases.^15^ This capability is especially important with RNA-based therapeutics, as RNA is less stable than its DNA counterpart, although recent backbone and base modifications have improved this.^16^ Further, nanoparticles enhance the rate of cell uptake and reduce clearance, but the precise mechanism of uptake will depend on particle size, shape, and surface chemistry.^17,18^ ASO-SNA core chemistry has also been studied and it was found that hollow, cross-linked SNAs and molecular SNAs have comparable cell uptake properties to traditional inorganic core (i.e. AuNP) SNAs while maintaining functional antisense activity.^19−21^ Self-assembled SNAs to deliver ASOs have been developed using hydrophobic cores to provide facile synthesis while maintaining the benefits of nanoparticle-mediated delivery.^22−25^ Typically, nanoparticles, including SNAs, invoke passive targeting strategies to overcome extracellular barriers and reach a biological target; however, nanoparticle cargo often remains trapped within endosomes and is degraded before it escapes to enact a therapeutic response.^15^ These passive targeting strategies impose large nanoparticle concentrations or inert surface modifications to increase blood circulation time.^26^ Stimuli-responsive SNAs are being actively developed to enable controlled release of ASO payloads, yet these SNAs either require extracellular triggers or lack efficient cytosolic delivery.^25,27,28^

One general strategy to efficiently deliver nucleic acid drugs into the cytosol is to enhance leakage or escape from endosomes. This typically involves the use of membrane-active agents. For example, cationic amphiphilic drugs such as chloroquine, siramesine, and bafilomycin disrupt the endosomal maturation pathway by preventing acidification and increasing nonspecific escape.^29−31^ Other small molecules such as Triton X-100 monomer and amphotericin B function through direct membrane disruption and inducing increased endosomal leakage.^32^ Other studies have shown that alternative delivery strategies such as attenuated diphtheria toxin trafficking for siRNA can increase cytosolic delivery.^33^

Similarly, researchers have developed peptides and proteins that can also induce endosomal escape.^34−37^ This class of peptides, often named cell-penetrating peptides (CPPs) or endosomal escape peptides (EEPs), are typically cationic or amphipathic and disrupt membranes efficiently.^35,38−44^ While many cationic peptides are membrane active, the lack of specific endosomal activity is associated with toxicity and has hindered clinical translation.^32,45,46^ A recent siRNA delivery strategy used a pH-sensitive acetal group to trigger the release of a caged surfactant to disrupt the endosome; however, this strategy is limited by poor stability of the acetal group at physiological pH and requires high concentrations to disrupt the endosome.^47^ Others have reported optically controlled endosomal escape using aggregation induced emission photosensitizer nanoparticles.^28,48^ Although these site-targeted strategies greatly decrease nonspecific disruption, poor light penetration to deep tissue limits human translation as a platform technology.

Interestingly, influenza viruses are ∼100 nm particles that are efficiently taken up by endocytosis, but have evolved a stealthy strategy to escape endosomes and release their genetic content. Hemagglutinin proteins on the surface of influenza bind its sialic acid targets in the endosome and then undergo a massive conformational change that disrupts the endosome but only upon acidification.^49^ In this way, hemagglutinin is effectively “spring loaded” and biophysically disrupts the endosomal membrane upon acidification. We were inspired by this mechanism to develop an ASO membrane disruption agent that is released selectively upon acidification, thus minimizing off-target effects.

We report the development of an ASO delivery system termed **D**NA **E**ndosoma**L** Escape **V**ehicle **R**esponse (DELVR) that senses and responds to the nuclease-rich and acidic environment of the endosome to release its drug cargo. DELVR comprises two main components: the first is an ASO conjugated to an EEP, and the second is a nanoparticle coated with a pH-responsive complementary oligonucleotide anchor. In DELVR, tens of copies of the ASO-EEP are hybridized to nanoparticles modified with anchor oligos. The orientation is critical, and the EEP is designed to face the interior of the nanoparticle, thus concealing the EEP. Importantly, the anchor-ASO duplex is highly sensitive to acidic pH and nucleases that release the ASO-EEP drug. In this work, we optimize oligo sequences and EEP composition to achieve a maximal pH response and identify conditions for enhanced ASO activity. By benchmarking DELVR against that of conventional nanoparticle ASO’s and conventional ASO-EEP conjugates, we demonstrate that this platform offers enhanced efficacy and potential for boosting the activity of validated ASOs in clinical development.

## Results and Discussion

**Scheme 1:**
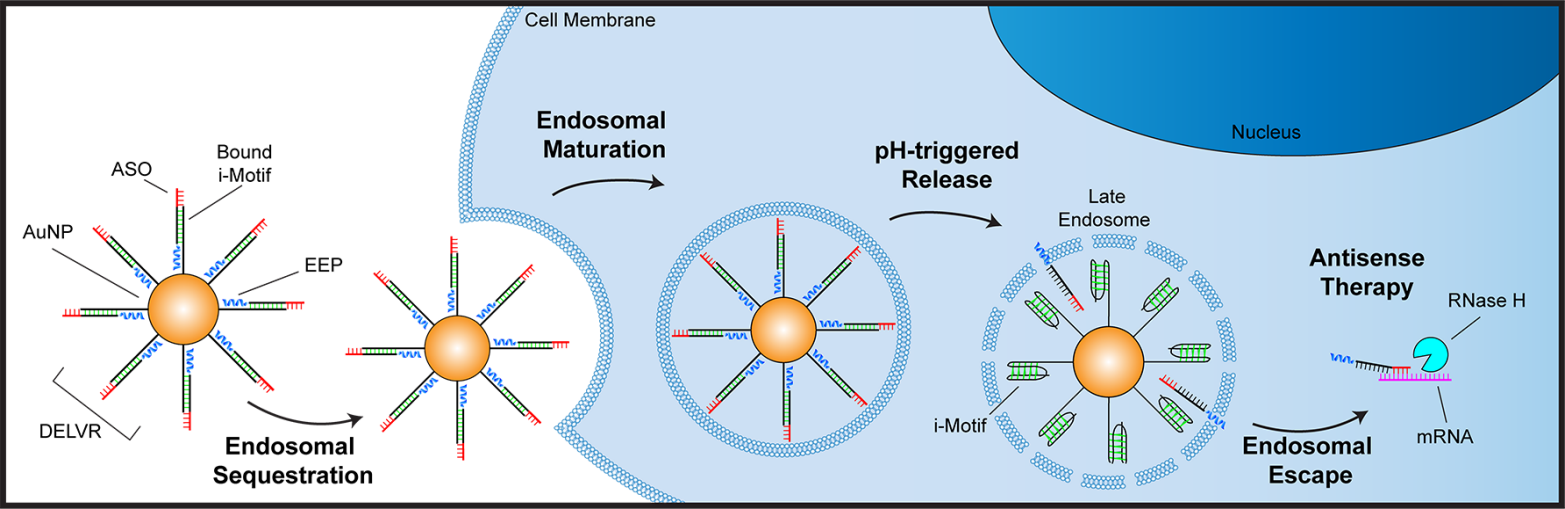
Cellular Mechanism of DELVR DELVR is composed of a nanoparticle core (AuNP) bound directly to an i-Motif strand (black with base pairing in green) and is hybridized to a complement strand containing an antisense oligonucleotide (ASO in red) and endosomal escape peptide (EEP in blue). DELVR is taken up into the cell within endosomes via endocytosis. As the endosome matures, the i-Motif strand on DELVR responds to the acidification, causing release of the complement and exposing the membrane-active EEP. The EEP selectively disrupts the endosomal membrane, leading to endosomal escape of the ASO and allowing for antisense therapeutics to reach their targets.

The main objective of DELVR is to circumvent the endosomal entrapment of nucleic acid drugs, as it is well-documented that the vast majority of nanoparticles and nucleic acid therapeutics are entrapped within endosomes.^50^ Our strategy is summarized in Scheme 1. Briefly, a blocked (double-stranded) ASO that is tethered to a nanoparticle is delivered to cells, where the conjugates are internalized by endocytosis. Upon endosomal maturation, the pH of the endosome drops and acts as the primary trigger for the release of the ASO from the AuNP by dissociation from the i-Motif oligonucleotide anchored to the AuNP. Nucleases also act as a secondary release mechanism for the ASO-EEP cargo. The release activates the ASO and exposes the EEP to disrupt the endosomal membrane, thus allowing for active delivery into the cell cytoplasm.

**Figure 1:**
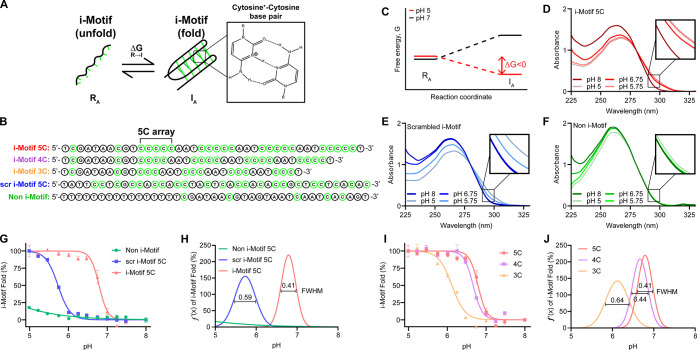
Characterization of i-Motif pH response. A. Schematic representing i-Motif folding. R_A_ and I_A_ refer to random coiling and i-Motif structures. Zoom-in image shows the hemiprotonated cytosine to cytosine hydrogen bonding. B. Sequences for each DNA strand tested in this figure with names corresponding to the size of repeated cytosine arrays or the distribution of cytosines (cytosines are highlighted in green). C. A proposed free energy diagram showing that the transition from single-stranded DNA (ssDNA) to i-Motif is spontaneous under acidic conditions and not favorable at neutral pH. Note that this diagram is hypothetical. D. The absorbance spectra for i-Motif DNA with arrays of 5 cytosines as a function of pH. E. The absorbance spectra for scrambled i-Motif DNA 5C as a function of pH. F. The absorbance spectra for non-i-Motif-forming DNA as a function of pH. G. Plot showing the percentage of DNA folded into an i-Motif versus pH for i-Motif 5C, scrambled i-Motif 5C, and non-i-Motif DNA. H. Plot showing the first derivative function of the i-Motif transition for i-Motif 5C, scr i-Motif 5C, and non-i-Motif DNA. I. Plot showing the i-Motif folded percentage vs pH for i-Motif 3C, 4C, and 5C. J. Plot showing the first derivative function of i-Motif transition for i-Motif 3C, 4C, and 5C DNA. For G and I, plots were fitted to Boltzmann sigmoidal distributions to determine the p*K*_a_. For H and J, the first derivative of the i-Motif transition was calculated to determine the FWHM profile of each DNA group. Experiments were conducted in triplicate with 5 μM DNA concentration in 1× UB4 buffer (157 mM Na^+^ with 0 M Mg^2+^).

To develop the pH-triggered release mechanism, we chose to use i-Motif DNA, as it is well documented to fold in acidic environments.^51−53^ When acidified, cytosine nucleobases become hemiprotonated and can form hydrogen bonds to another cytosine nucleobase (Figure 1A). This is a noncanonical Watson–Crick base pairing interaction and leads to the development of a four-stranded antiparallel structure, called an i-Motif. To explore this folding process, we screened three i-Motif sequences with increasing C-tract length, a scrambled i-Motif sequence, and a non-C-rich sequence (Figure 1B, Table S1). Folding into the i-Motif structure is thermodynamically favored at increasing H^+^ concentration (Figure 1C).^54,55^ Guided by literature precedent, we designed i-Motif sequences that displayed a repeating pattern of 3, 4, or 5 C-bases separated by AAT spacers.^56,57^ We first quantified i-Motif folding of a sequence containing tracts of 5 C-bases (40% C-bases) by UV–vis spectroscopy, which showed a bathochromic and hypochromic shift upon acidification (Figure 1D). Importantly, when this sequence was scrambled, the response was highly dampened (Figure 1E), thus confirming that the repeating C-base pattern was central for i-Motif folding. The C-bases are critical to enable pH response, as a control sequence with 12% C-base composition showed no observable chromic shift (Figure 1F). Using absorbance at λ = 295 nm, a unique i-Motif absorbance signature,^51,58^ we calculated the percentage of DNA folded into the i-Motif structure as a function of pH (Figure 1G). Data were normalized using pH 5.0 and pH 8.0 as the 100% and 0% folded values, respectively. By fitting the data to a Boltzmann sigmoidal function, we found that the transition pH (p*K*_a_) for 5 C-tract i-Motif and scrambled i-Motif were 6.79 (±0.020) and 5.73 (±0.011), respectively (Figure 1G and 1H). The non-i-Motif DNA failed to show a detectable transition within the pH range tested. We also found that the pH transition was highly dependent on the number of C-bases in a row as the 3, 4, and 5 C-tracts displayed transition pH values of 6.12 (±0.023), 6.67 (±0.016), and 6.79 (±0.020), respectively (Figure 1I). This validates the role of C-bases in stabilizing folding of the i-Motif structure.^53,59^ To further characterize i-Motif folding, we calculated the first derivative for each i-Motif pH transition and used the full width at half-maximum (FWHM) of the transition to determine the sharpness of the transition. We found that i-Motif 5C has a FWHM of 0.414 pH units, whereas the scrambled i-Motif FWHM was 0.585 pH units (Figure 1H). Additionally, the structured i-Motif 3C, 4C, and 5C were found to have FWHM values of 0.640, 0.436, and 0.414 pH units, respectively, confirming that increasing the number of C-bases in a row leads to a narrowing of the pH transition profile (Figure 1J). Finally, given that these oligos are being designed with *in vivo* applications in mind, we also sought to quantify the pH transition for the nuclease-resistant phosphorothioate (PS)-linked nucleic acids. We found that PS-modified 5C–i-Motif sequences showed a pH transition of 6.83 (±0.023) and FWHM of 0.919 pH units (Figure S1). Importantly, the observed lack of shift in i-Motif p*K*_a_ for the PS modification agrees with prior literature, while the broadening of the FWHM is likely the result of the racemic mixture found in PS DNA.^60^

**Figure 2:**
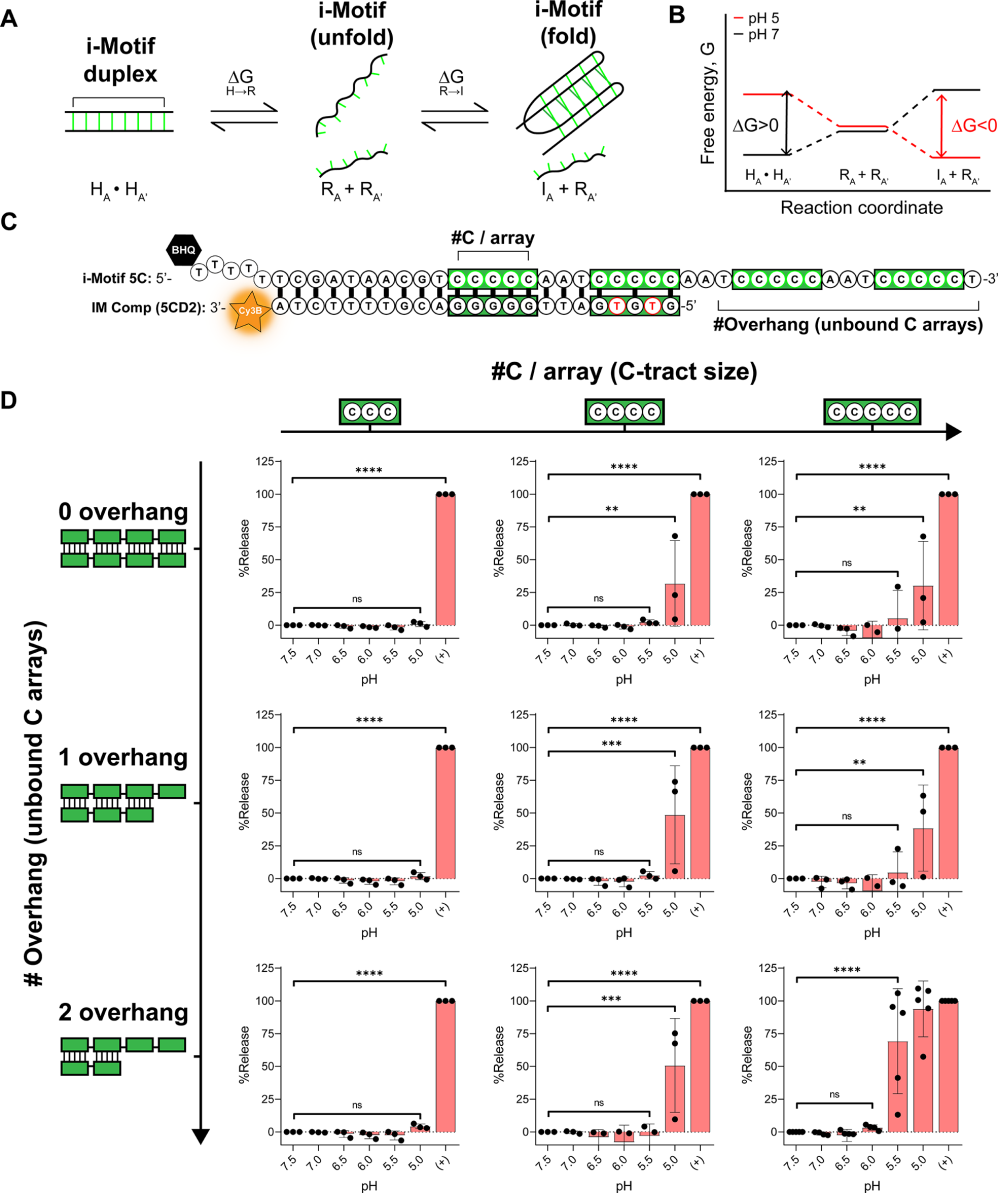
Screening identifies a structure-switching i-Motif duplex that is triggered at pH 5.5. A. Schematic representing the transition from double-stranded DNA (dsDNA) to folded i-Motif and random coil DNA. B. Proposed free energy diagram for the structure-switching i-Motif at neutral and acidic pH. Note that this diagram is hypothetical. C. Diagram showing the duplex design to screen the i-Motif trigger mechanism. The diagram specifically depicts the 5C-base i-Motif and the two C-tract overhang complement. Cy3B is a pH-insensitive fluorescence reporter that is quenched with a black hole quencher (BHQ). Intentional mismatches minimize the formation of G-quadruplexes and maintain equal melting temperatures. D. Screen quantifying duplex denaturation as a function of pH. Nine i-Motif duplexes were investigated as a function of # C-bases per array and # C-array overhangs. Each i-Motif contains four arrays, and the number of bound arrays varied with increasing overhang. Each individual trial is normalized to a thermally melted positive control (“+”) to determine % release as a function of pH. Experiments were conducted in at least triplicate at 52.5 nM quencher and 50 nM Cy3B strand for 3 h at 37 °C. One-way ANOVA tests were conducted with *post hoc* Tukey’s tests against the pH 7.5 group.

While single-stranded i-Motif structure switching has been extensively studied,^54,59,61,62^ designing an i-Motif that can switch from a duplexed state at neutral pH into a folded single-stranded state at acidic conditions is far less common.^63^ To create such a structure-switching i-Motif, the initial duplex must be stable at neutral pH and at 37 °C. Upon acidification, the duplex must dehybridize. Lastly, the i-Motif must fold into its tertiary structure and display stability at this acidic pH (Figure 2A). The process must be spontaneous under acidic conditions and highly unfavorable at neutral pH (Figure 2B). Multiple i-Motif duplex sequences were screened. A fluorescence quencher in proximity to the Cy3B fluorophore allowed for fluorescence reporting of duplex denaturation (Figure 2C). Additionally, intentional base pair mismatches were implemented to prevent the formation of the G-quadruplex, which can hinder duplex formation or downstream application (Table S1). All duplexes were designed with similar melting temperature (*T*_M_) so that thermal stability did not mask pH responsivity, while maintaining stability at 37 °C (Figure S2). We measured the fluorescence increase as a function of pH for the i-Motif library, exploring the C-tract density as well as the number of C-arrays that remained unbound by the duplex as an overhang (Figure 2D). Screen conditions and kinetics were optimized to reduce nonspecific release due to temperature or incubation duration (Figure S3). We discovered that as the overhang length increased, the p*K*_a_ slightly increased, as indicated by a shift in % release toward more neutral pH values, although this was not significant. Further, a significant increase in p*K*_a_ was observed with increasing C-tract length from three to five. A two-way ANOVA was conducted to quantify trends from overhang length as well as C-tract length for this screen (Figure S4). With these two trends, it was found that the best candidate for the i-Motif duplex trigger was the combination of the five C-tract length i-Motif and two array overhangs, which is termed the i-Motif 5C-5CD2 duplex from here on.

**Figure 3:**
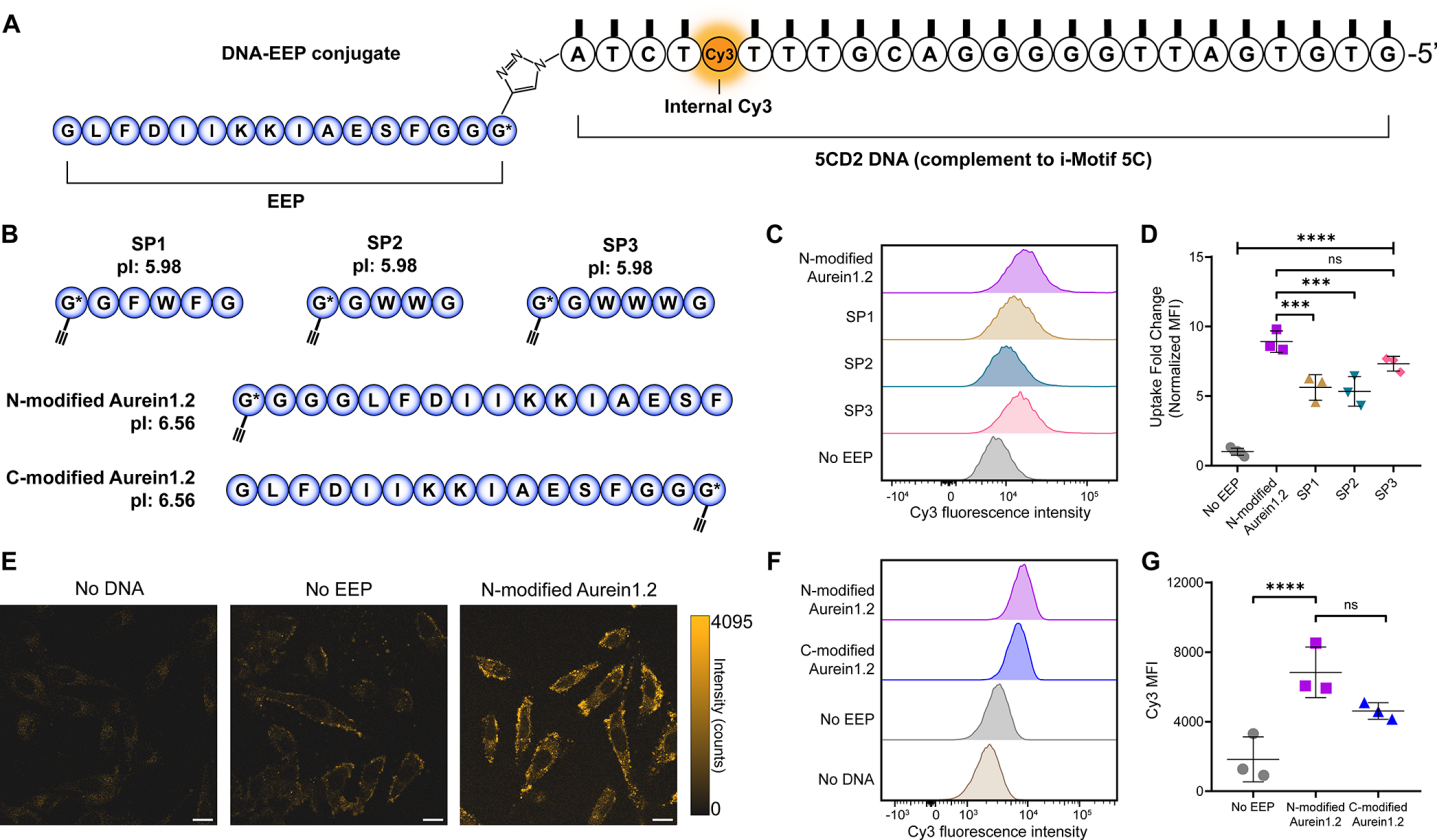
Endosomal escape peptides increase uptake for oligonucleotide conjugates. A. Schematic showing conjugation strategy between the oligonucleotide (5CD2) and endosomal escape peptide (EEP). The EEP contains an alkyne-modified glycine (propargylglycine: G*) at a terminus that reacts with a 3′ azide group on the DNA through a copper-catalyzed azide–alkyne cycloaddition reaction (copper click). The oligonucleotide contains an internal Cy3 modification to enable fluorescence reporting. B. Schemes showing peptide sequences for EEP used in this study (SP1, SP2, SP3, N-modified Aurein1.2, and C-modified Aurein1.2). The isoelectric points (pI) are listed as well. C. Flow cytometry histograms showing distribution of Cy3 fluorescence uptake in HeLa cells among N-modified Aurein1.2, SP1, SP2, SP3, and a no EEP negative control. HeLa cells were incubated with 50 nM DNA-EEP for 4 h in serum-free media. D. Plot indicating uptake fold change with N-modified Aurein1.2, SP1, SP2, and SP3 EEPs, and no EEP DNA conjugates. Each group was measured in triplicate and background subtracted against untreated cells. A one-way ANOVA test was used to compare each group with *post hoc* Tukey’s tests comparing each group to Aurein1.2. Points represent independent trials, and error bars represent standard deviation. E. Representative confocal images of EEP uptake after 1 h of incubation with 250 nM DNA-EEP labeled with Cy3. Scale bar = 20 μm. F. Flow cytometry histograms showing the distribution of Cy3 fluorescence in HeLa cells between C-modified and N-modified Aurein1.2 compared against the negative controls (no EEP and no DNA). HeLa cells were incubated with 50 nM DNA-EEP for 4 h in serum-free media. G. Plot comparing the Cy3 MFI across different EEP orientations. Fluorescence is background subtracted from the untreated group and compared using a one-way ANOVA with *post hoc* Tukey tests against the negative control. Points represent independent trials, and error bars represent standard deviation. Note: E–G were measured with 5CD2 containing a 5′ Cy3 dye rather than internal Cy3 modification. *P* values are reported as ns (*P* > 0.05), * (*P* < 0.05), ** (*P* < 0.01), *** (*P* < 0.001), and **** (*P* < 0.0001).

A representative structure of an oligonucleotide conjugated to Aurein1.2, which is an antimicrobial peptide isolated from *L. aurea* skin and is well documented for endosomal escape properties, is shown in Figure 3A. One challenge with using EEPs is that positively charged amino acids can nonspecifically interact with the negatively charged DNA backbone. To address this potential problem, a small screen of short amphipathic EEP-DNA conjugates was conducted to explore the activity of other EEPs in comparison to that of Aurein1.2 (Figure 3B, Table S2).^100^ Overall, it was found that the conjugation of EEPs to DNA significantly increased cellular uptake in HeLa cells, as measured by flow cytometry, where the Cy3 signal associated with the conjugate was quantified (Figures 3C,D, S5). Additionally, to confirm that the conjugates were entering cells, confocal microscopy visualized the uptake process and revealed that in addition to increased Cy3 fluorescence signal from each cell, the EEP-DNA fluorescence was more evenly distributed across the cell, suggesting endosomal escape rather than general membrane association (Figure 3E). Furthermore, EEP orientation also influences cellular uptake as N-modified Aurein1.2 significantly improved uptake in HeLa cells, whereas C-modified Aurein1.2 did not significantly improve uptake compared to the nonmodified oligonucleotides (Figure 3F,G). Note that Figure 3E–G were measured using a 5′ Cy3 modification rather than internal Cy3 modification (Table S1). To further understand how EEP orientation affects cellular uptake, we utilized AlphaFold2 to predict the terminally modified Aurein1.2 structures and found that the C-modified Aurein1.2 has more hydrophobic residues exposed at the non-DNA-conjugated end, while the N-modified Aurein1.2 exposure is more hydrophilic (Figure S6). Previous reports show that Aurein1.2 functions through carpet mechanism disruption via interactions of hydrophobic and hydrophilic residues with lipid membranes.^64^ As such, a terminal modification of an anionic and hydrophilic DNA molecule would influence the Aurein1.2 function. As the N-modified Aurein1.2-DNA conjugate showed the highest level of HeLa cell uptake as indicated by flow cytometry and confocal microscopy, EEP will refer to N-modified Aurein1.2 from here on unless otherwise noted.

**Figure 4:**
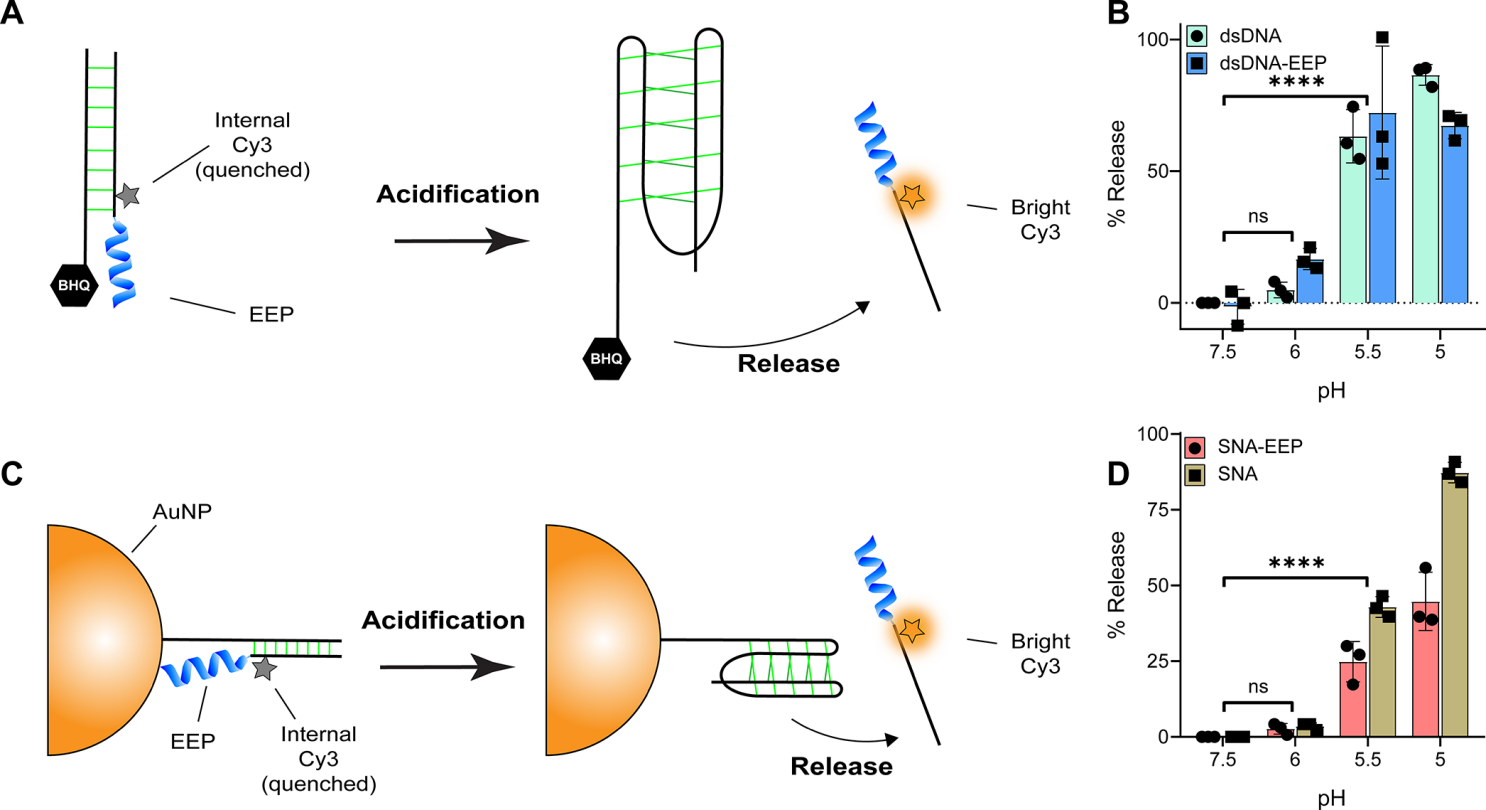
Evaluation of the pH response for structure-switching i-Motif duplex with EEP and on AuNP. A. Scheme showing i-Motif duplex quenching mechanism with EEP. The internal Cy3 allows for FRET reporting of duplex dissociation upon acidification. B. Quantification of duplex release between i-Motif 5C and its complement (5CD2) with or without EEP at varying pH. 52.5 nM quencher strand (i-Motif 5C) and 50 nM Cy3-EEP strand (5CD2) were annealed and subsequently incubated together for 3 h at 37 °C in varying pH buffer (1× UB4). Samples were then fluorescently measured via a plate reader. C. Scheme showing the i-Motif AuNP release mechanism using the AuNP as an NSET quencher. D. Quantification of duplex release on AuNP between i-Motif 5C and its complement (5CD2) with or w/o EEP at varying pH. Constructs were incubated for 3 h at 37 °C in varying pH buffer (1× UB4) and then fluorescently measured via plate reader. For B and D, the % release is normalized to a thermally melted positive control, which indicates complete release. Statistics were performed using one-way ANOVA tests and *post hoc* Tukey tests comparing each group to pH 7.5. *P* values are reported as ns (*P* > 0.05), * (*P* < 0.05), ** (*P* < 0.01), *** (*P* < 0.001), and **** (*P* < 0.0001). All groups were measured in triplicate.

To realize the DELVR concept, we next explored the ability of the i-Motif DNA-EEP conjugate to function on a SNA. First, we tested the effect of EEP conjugation to DNA in the i-Motif pH response. The i-Motif 5CD2 with terminal Iowa Black quencher was hybridized to the EEP conjugated to its counterpart with an internal Cy3 (Figure 4A). Through FRET, Cy3 fluorescence is quenched when i-Motif 5CD2 is hybridized. Upon acidification, i-Motif 5CD2 folded and then released the N-terminal Aurein1.2 conjugated complement, dequenching the Cy3 fluorescence signal. We validated that the double-stranded duplex maintains its response to acidification in the presence of the EEP (Figure 4B). This shows that the electrostatic interaction between the EEP and DNA does not significantly alter the pH response. Similarly, we tested the i-Motif function on a spherical nucleic acid. The i-Motif was anchored to a gold nanoparticle core through a thiol–gold interaction (Figure 4C). This i-Motif anchor was hybridized to the 5CD2 oligo modified with an internal Cy3 as well as a N-modified Aurein1.2 EEP oriented toward the AuNP core. We found that each AuNP contained 164.9 (±5.6) i-Motif anchor strands and 91.7 (±9.4) complement strands as measured by OliGreen and Cy3 reporter assays (Figure S7). Note that less than 100% hybridization efficiency is expected with dsDNA-SNAs and that the highest hybridization efficiency is achieved using the freeze method to synthesize SNAs, which was utilized within this paper.^65,66^ Through nanometal surface energy transfer (NSET), the Cy3 was quenched when DELVR was fully intact at neutral pH; however, once acidification occurs, the complement is released, allowing Cy3 to recover fluorescence. When we tested the release on the gold core, pH responsivity was maintained, but the total amount of oligo released was reduced by 36% when compared to that of the soluble duplex. This suggests that the EEP may interact with the gold core, hindering full release (Figure 4D). Peptide interaction with the surface of AuNP is well documented and is consistent with our findings.^67,68^ Another potential complication pertains to the molecularly crowded cellular environment that could hinder the i-Motif duplex release. To emulate this environment, we tested how PEG-8K at densities ranging from 5% to 20% mass/mass impacted the p*K*_a_ and % release of oligonucleotides from the AuNP core (Figure S8). We found that the 20% PEG-8k concentration led to a small <10% shift in p*K*_a_ of the i-Motif duplex and % release. This suggests that the cell environment will slightly alter the DELVR pH response.

We next designed a series of experiments to test the efficacy of DELVR *in vitro*. The general mechanism for ASO drugs is shown in Figure S9a. We chose hypoxia inducible factor 1a (HIF1a) as our target, as it is upregulated in hypoxic tissues, often associated with solid tumor-forming cancers. The ASO (EZN2968) used in this study is well-established *in vitro* and was tested as an antitumor agent in two clinical trials, but has not progressed to FDA approval.^69^ Accordingly, we decided to work with this ASO given the potential to enhance its activity and catalyze its progression as an efficacious therapeutic. We confirmed the activity of this HIF1a-targeting ASO in HeLa cells by measuring HIF1a mRNA expression using RT-qPCR with 18S as a housekeeping gene (Figure S9B,C). We found that the ASO is potent when delivered using oligofectamine transfection agent (EC_50_ ∼10 nM) (Figure S9C); however, the efficacy decreases with only the soluble DNA (Figure S9D). In both cases, it maintains a concentration-dependent response, indicating that the drug can target HIF1a. Additionally, as DELVR requires direct hybridization to the i-Motif anchor, it was found that the binding region did not affect the ASO efficacy, as the ASO is a gapmer design and will bind mRNA with higher specificity due to locked nucleic acid modifications (Figure S9E).

**Figure 5:**
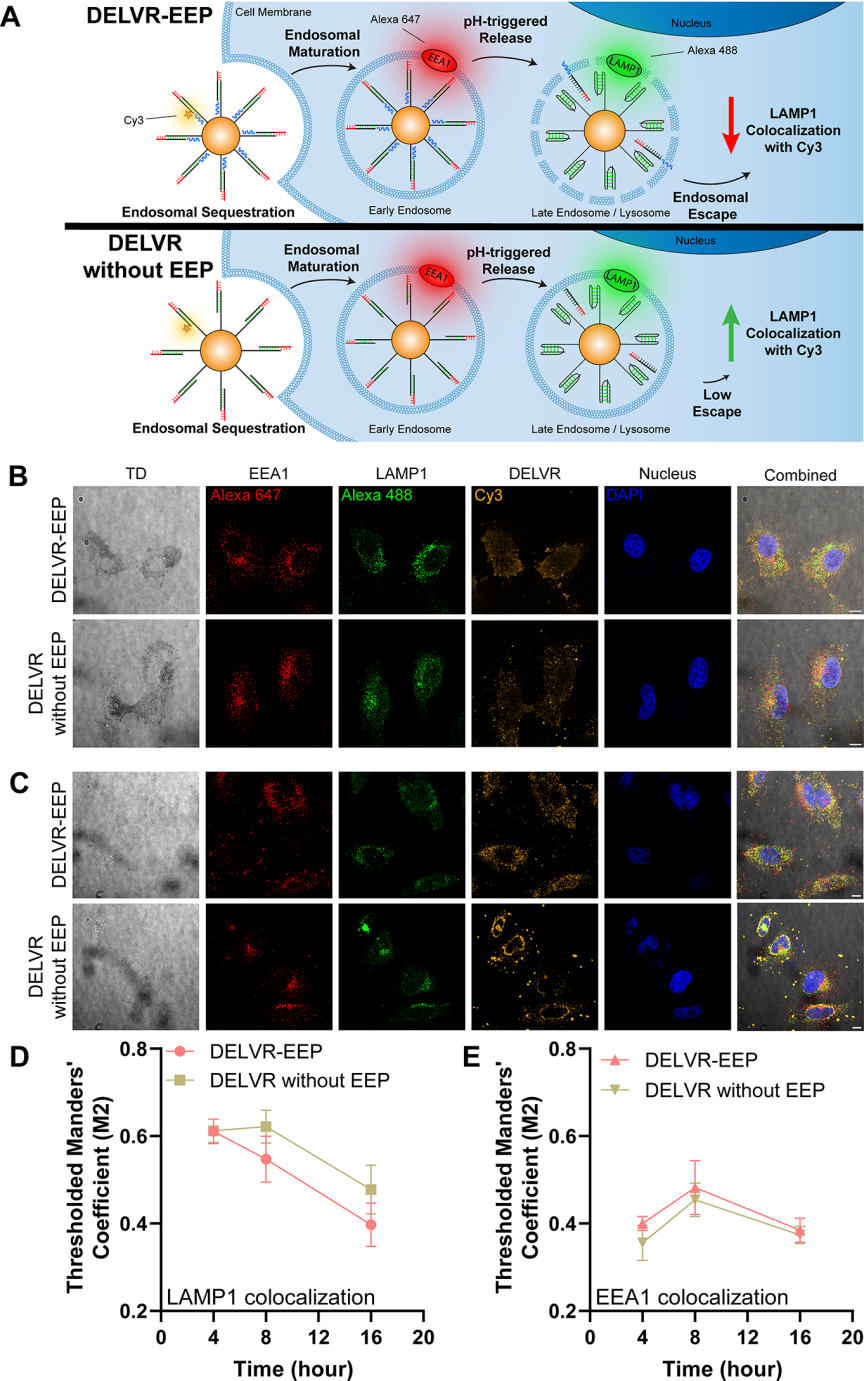
Visualization of DELVR colocalization with endosomes. A. Schematic showing endosomal entrapment of DELVR containing EEP (N-modified Aurein1.2, top) and DELVR without EEP (bottom). Early endosomes are labeled through anti-EEA1 staining conjugated to Alexa Fluor Plus 647 dye. Late endosomes and lysosomes are labeled through anti-LAMP1 staining conjugated to Alexa Fluor Plus 488 dye. DELVR-EEP escapes within late endosomes and lysosomes as indicated by decreasing colocalization of Cy3 signal to the LAMP1-Alexa488 signal. B. Multichannel fluorescent confocal images of DELVR-EEP (5 nM) and DELVR without EEP (5 nM) after incubation for 4 h in HeLa cells. C. Multichannel fluorescence confocal images of DELVR-EEP (5 nM) and DELVR without EEP (5 nM) after incubation for 8 h in HeLa cells. In B and C, HeLa cells were fixed and permeabilized before labeling with monoclonal primary and fluorescently tagged secondary antibodies. The TD (gray), early endosome (red), late endosome (green), DELVR (orange), nucleus (blue), and combined channels are shown. Scale bars = 10 μm. Note that images were collected as a Z-stack despite one representative slice shown above. D. Plot showing thresholded Manders’ coefficient M2 (Cy3 signal overlapping LAMP1 signal) for both DELVR-EEP (light red) and DELVR without EEP (gold). E. Plot showing thresholded Mander’s coefficient M2 (Cy3 signal overlapping EEA1 signal) for both groups. Groups were measured in at least triplicate.

To confirm DELVR’s endosomal escape potential, colocalization analysis using known endosomal markers enables us to understand cellular distribution with high precision. To accomplish this, we incubated HeLa cells with 5 nM DELVR containing EEPs or 5 nM DELVR without EEPs for 4, 8, or 16 h. These cells were fixed, permeabilized, and antibody stained to label early endosomes (EEA1), late endosomes/lysosomes (LAMP1), and nuclei (DAPI) (Figure 5A). We collected Z-stack confocal images for multiple fluorescent channels to reduce imaging bias and enable quantification of colocalization (Figure 5B,C). It was hypothesized that both groups would have similar colocalization with EEA1, while a difference in LAMP1 colocalization would indicate effective endosomal escape, as DELVR is pH-responsive to values found in late endosomes or lysosomes. Qualitatively, the DELVR-EEP Cy3 signal appears more confluent in both the 4 h (Figure 5B) and 8 h incubation (Figure 5C) compared to DELVR without EEP. Further, Cy3 signal from DELVR without EEP appears punctate in both the 4 h incubation (Figure 5B) and 8 h incubation (Figure 5C) with additional localization around the nuclear edge. For quantification via colocalization analysis, we employed automatic Costes’ thresholding across the entire Z-stack for each individual cell, and we chose Manders’ coefficient (M2) to evaluate the DELVR Cy3 signal overlapping the EEA1 or LAMP1 signal. We found that the two DELVR groups shared similar LAMP1 M2 values at 4 h; however, after 8 h incubation, colocalization of DELVR-EEP with LAMP1 decreased, while DELVR without EEP slightly increased (Figure 5D). This shows that EEP enables DELVR to escape from endosomal vesicles, rescuing the construct from lysosomal degradation. After 16 h, LAMP1 colocalization decreases for both groups, indicating nonspecific leakage and/or degradation of DNA, with DELVR-EEP having the lowest colocalization value (Figure 5D). Studying EEA1 colocalization, we found that both DELVR-EEP and DELVR without EEP share similar M2 values at 4, 8, and 16 h of incubation, suggesting that DELVR is not responsive to early endosomal conditions as predicted (Figure 5E).

**Figure 6:**
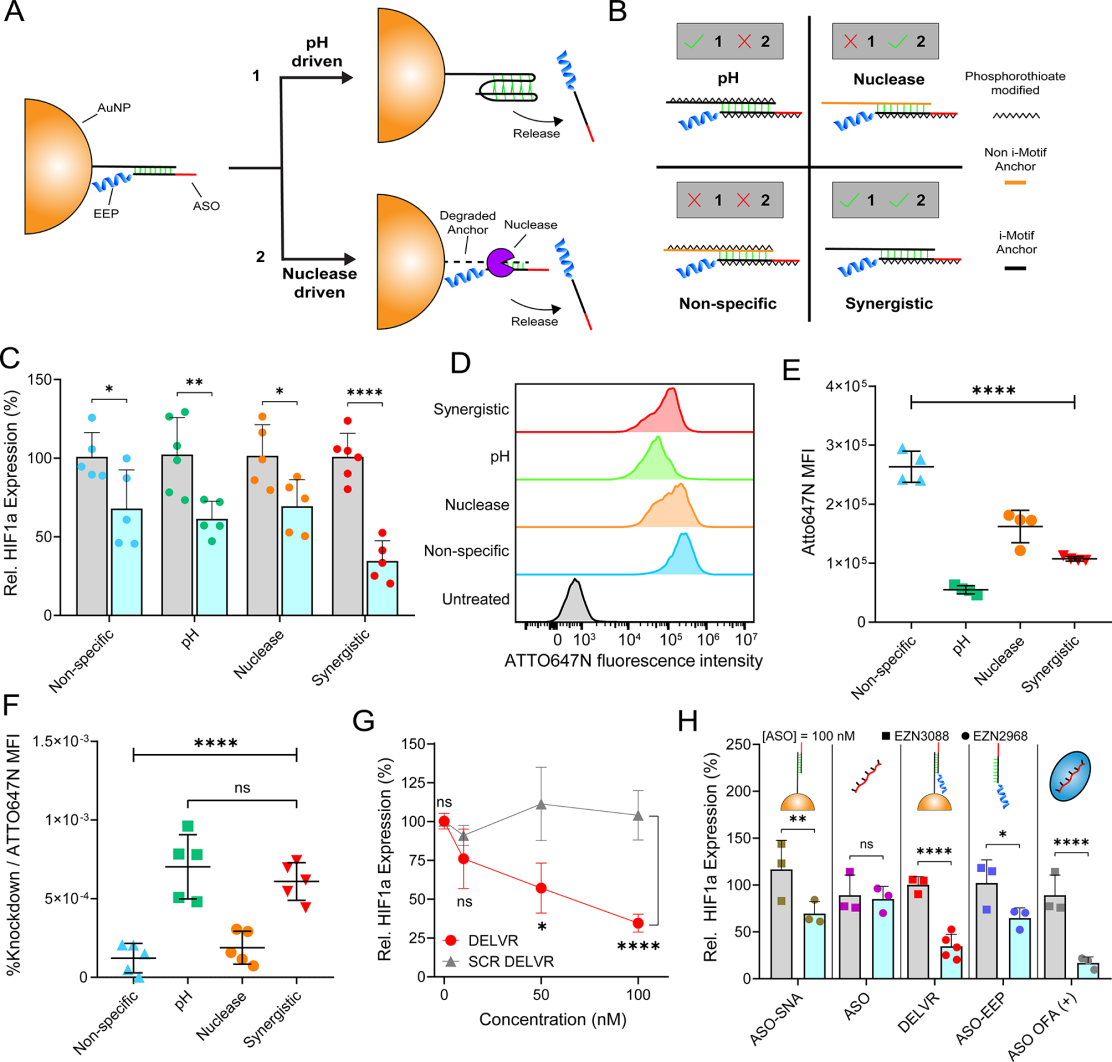
Evaluation of DELVR efficacy and cellular release mechanism. A. Scheme showing potential release mechanisms of DELVR, invoking pH or nuclease-driven cues. Nucleases can bind and degrade dsDNA in lysosomes. B. Scheme showing release mechanism screen for DELVR. The check mark or × indicates release mechanism susceptibility, with colors indicating anchor pH responsivity. The spikes represent PS linkages. Note the complement contains PS linkages, EEP, and ASO in all designs. C. Relative HIF1a knockdown across each release group was observed at 100 nM ASO (EZN2968). Data are normalized against untreated and scrambled ASO (EZN3088) DELVR negative controls. D. Flow cytometry histograms of ATTO647N uptake for each release group and negative control in HeLa cells after 1 h at 100 nM dye-complement. E. HeLa cell uptake through the ATTO647N MFI for each release group. Data are background subtracted with negative control. F**.** Normalization of %HIF1a knockdown per ATTO647N MFI was performed for each release group. This measures the endosomal escape efficiency. G. DELVR dose dependence (synergistic group) to knockdown HIF1a at 0, 10, 50, and 100 nM ASO compared to that of scrambled ASO DELVR with 100.3%, 76.1%, 57.2%, and 34.6% HIF1a expression, respectively.Statistics represent comparisons against the respective scrambled control for each concentration with error bars representing SEM. H. Antisense knockdown and comparison of each modular DELVR component. Statistics were performed using a two-way ANOVA with *post hoc* Fisher’s LSD tests. Statistics were compared to the scrASO equivalent (EZN3088). In C, G, and H, HIF1a knockdown is normalized using the ΔΔCt method with the scrASO and 18S housekeeping gene. *P* values are reported as ns (*P* > 0.05), * (*P* < 0.05), ** (*P* < 0.01), *** (*P* < 0.001), and **** (*P* < 0.0001). Individual comparisons are indicated with a bar. All groups were measured in at least triplicate with error bars representing SD unless noted. Note that concentration refers to oligonucleotide concentration (ASO, not AuNP), and ASO concentration was measured through use of 10 mM KCN to etch AuNP and measure complement oligonucleotide concentration before experimentation.

Having confirmed that ASO was active, we next investigated whether DELVR could enhance its efficacy. Given the highly acidic and nucleolytic environment of endosomes, we postulated two nonexclusive mechanisms for ASO activation (Figure 6A): pH-driven and nuclease-driven. Accordingly, we designed four DELVR constructs to help explore these mechanisms of action. These constructs are shown in Figure 6B and included oligos that were pH-responsive or nuclease-sensitive. Note that the anchor strand complement was fully PS modified and contained LNA modifications to reduce nuclease activity. The pH DELVR construct used a PS modification to diminish nuclease-driven release of the ASO, while maintaining pH activity. In contrast, the nuclease DELVR contained a PO backbone but lacked the i-Motif sequence and hence primarily released due to nuclease action. The nonspecific DELVR had a PS backbone and lacked the i-Motif and thus served as a control. Finally, the synergistic DELVR contained both a PO backbone and i-Motif and responded to both DNase and pH inputs. When we incubated 100 nM of the four DELVR constructs with HeLa cells for 24 h, we found that the synergistic DELVR showed a significant improvement in HIF1a knockdown compared to the three other groups (Figure 6C). This confirms the optimal design for DELVR and shows that the mechanism of action functions through a combination of both nuclease and pH activity. Moreover, to confirm that DELVR is an effective endosomal escape platform, we quantified HeLa cell uptake through flow cytometry, measured using ATTO647N fluorescence (Figure 6D). To ensure that DELVR constructs remain intact and mean fluorescence intensity (MFI) was proportional to HeLa cell uptake, HeLa cells were briefly incubated with the four DELVR constructs for 1 h. We found that the pH and synergistic DELVR constructs had the lowest cellular uptake, while the nuclease and nonspecific DELVR constructs were highest (Figure 6E). By normalizing cellular uptake to antisense knockdown activity, we found that pH-sensitive DELVR constructs (pH and synergistic constructs) were the most efficient antisense and endosomal escape therapeutics, validating DELVR as a delivery platform (Figure 6F). Finally, we measured the dose-dependent response of DELVR and show that DELVR is efficacious with an EC_50_ of 54.2 nM (Figure 6G). Furthermore, DELVR significantly outperforms the clinically tested drug, which is bare ASO in solution, and is comparable to the activity of ASO delivered by transfection agents (oligofectamine, OFA), which cannot be used *in vivo* due to toxicity (Figure 6H). The superior activity of DELVR (ASO-SNA-EEP) is demonstrated when measuring HIF1a knockdown efficacy compared to that of ASO-EEP (no AuNP) as well as ASO-SNA (no EEP) conditions, which are not as effective when statistically compared against their respective scrambled controls (Figure 6H).

**Figure 7:**
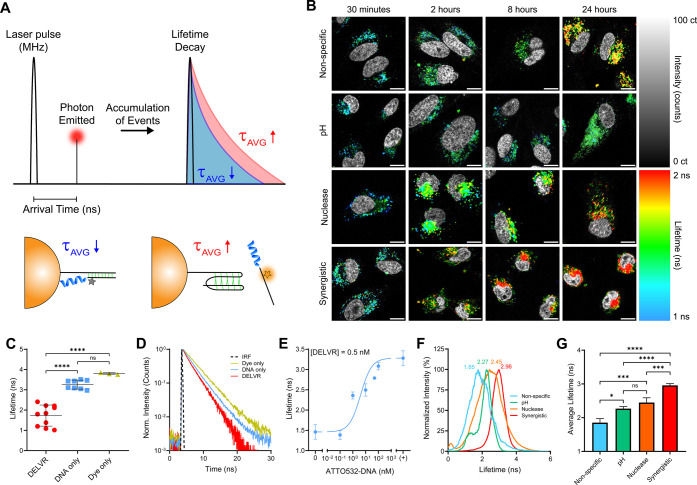
Quantifying DELVR triggering in cells using fluorescence lifetime imaging microscopy (FLIM). A. Schematic showing general FLIM principle, involving a pulsed laser (MHz) to collect an accumulation of emitted photon arrival times from a sample. Intact DELVR yields short lifetimes, and released DELVR yields long lifetimes. B. Intensity-weighted FLIM images of DELVR incubated within HeLa cells for various timeframes. DELVR constructs (2 nM) were administered to cells for 30 min before washing and then were imaged at the end of the incubation period. Blue indicates short lifetimes, and red indicates long lifetimes. A nuclear stain is overlaid on each image for colocalization in grayscale. Scale bar: 15 μm. C. Quantification of the average lifetimes for 0.5 nM intact synergistic DELVR, 100 nM ATTO532-DNA, and 100 nM unconjugated ATTO532 dye in 1× PBS. D. Lifetime decay curve for intact synergistic DELVR, ATTO532-DNA, and unconjugated ATTO532 dye with the corresponding measured instrument response function (IRF) using quenched erythrosine B. E. Average lifetime of a titration of unbound ATTO532-DNA to 0.5 nM synergistic DELVR constructs in 1× PBS. Data are represented by increasing concentration of unbound ATTO532 DNA. F, G. Average lifetime quantification for all four DELVR constructs after an 8 h incubation in HeLa cells using a biexponential decay model. The histograms represent an average pixel-wise distribution of intensity-weighted lifetimes and are normalized to the minimum and maximum intensity values of each condition. Error bars represent SD. All experiments were conducted in at least triplicate. Biological replicates were averaged across at least 30 cells and at least three ROIs to minimize imaging bias. Statistical comparisons were conducted using a one-way ANOVA with *posthoc* Tukey’s tests for individual comparisons (bar). *P* values are reported as ns (*P* > 0.05), * (*P* < 0.05), ** (*P* < 0.01), *** (*P* < 0.001), and **** (*P* < 0.0001).

Lastly, fluorescence lifetime imaging microscopy (FLIM) was conducted to visualize and quantify the DELVR *in vitro*. FLIM, compared to other fluorescence imaging techniques, provides a unique advantage for nucleic acids and nanomedicine, as fluorescence lifetime is a concentration-independent property of fluorophores that is dependent on its local environment.^70,71^ Here, we employ a pulsed laser to collect an accumulation of lifetime events from the sample based on emitted photons (Figure 7A). Utilizing NSET quenching interactions between ATTO532 and the AuNP, ATTO532 exhibits a short lifetime when bound within DELVR and a longer lifetime when released off the AuNP (Figure 7A–C). To quantify this interaction, intact synergistic DELVR constructs, ATTO532-conjugated 5CD2 DNA, and free ATTO532 dye were measured in 1× PBS buffer and found to have average lifetimes of 1.73 (±0.52), 3.26 (±0.22), and 3.80 (±0.05) ns (Figure 7C). This agrees with standard ATTO532 dye measurements provided by the manufacturer. The decay profile further corroborates this finding, as shown by a longer shift in lifetime decay (Figure 7D). To confirm the sensitivity of the FLIM measurement, we also titrated unbound ATTO532-DNA into a solution containing 0.5 nM DELVR to show that the lifetime increased as the ratio of unbound DNA to bound DELVR DNA increased (Figure 7E). As ATTO532 lifetime significantly increases once released off each DELVR, we incubated each DELVR construct in HeLa cells for various lengths from 30 min to 24 h to visualize each DELVR construct’s cellular release profile (Figure 7B). Indeed, we found that the shortest incubation times also exhibited the shortest lifetimes, quantified using a biexponential reconvolution decay model, as represented by blue-green colors (Figures 7B, S10). This indicates that each of the DELVR constructs is intact when entering cells and degrades over time as represented by the shift toward longer lifetimes (red color) (Figures 7B, S10). We found that after an 8 h incubation within HeLa cells, the synergistic DELVR had a significantly longer lifetime (τ_AVG_ = 2.97 ± 0.16 ns) compared to the nuclease DELVR (τ_AVG_ = 2.43 ± 0.10 ns), pH DELVR (τ_AVG_ = 1.92 ± 0.46 ns), and nonspecific DELVR (τ_AVG_ = 2.06 ± 0.31 ns) constructs, indicating a more rapid release of the SNA (Figure 7F,G). Interestingly, the pH and nonspecific DELVRs have the slowest release rates, likely stemming from the PS-modified backbone enhancing nuclease resistance. As a result, one explanation for synergistic DELVR’s rapid release may be that it follows a sequential two-part release with an initial nuclease-driven release followed by acid-responsive i-Motif release to enhance delivery. Additionally, a nuclear stain showed that the fluorescent signal was generated outside of the nuclear region, suggesting cytoplasmic delivery. This is further supported as longer incubation times display more confluent fluorescence rather than sparse and punctate distribution as found within endosomes (Figure 7B). Furthermore, the synergistic DELVR construct had the fastest release within cells with a half-life of 10.01 h^–1^, compared to pH (*t*_1/2_ = 22.83 h^–1^), nuclease (*t*_1/2_ = 15.26 h^–1^), and nonspecific release (*t*_1/2_ = 15.65 h^–1^) DELVRs, suggesting a potential explanation for the enhancement in knockdown efficacy compared to other constructs (Figure S10).

## Conclusion

In this work, we present a platform for improving the endosomal escape of nucleic acid therapeutics that is modular and can be used to boost drug efficacy of virtually any ASO. By first demonstrating the tunability of single-stranded i-Motif DNA, we show that i-Motif p*K*_a_ increases with structured cytosine repeats and increasing cytosine density, which led us to believe that i-Motif DNA may have further applications as a duplex release trigger. Our screen of i-Motif duplexes demonstrates the capability for i-Motif sequences to drive the denaturation of the duplex in response to acidification. We show that the duplex pair between the i-Motif with 5C repeats and its complement with an overhang of two C-arrays has an effective duplex release at pH 5.5, a release value well within the pH range present during endosomal maturation. These results demonstrate the capability of i-Motif duplexes to respond to an endosomal environment while maintaining physiological stability, which has previously limited i-Motif translation in drug delivery. Herein, we demonstrated an application of this i-Motif duplex sequence for antisense therapies, though this design can be applied more broadly for RNA interference therapies, nanoflares, and other cellular delivery applications for nucleic acids.

Membrane-active agents such as EEPs have performed poorly in clinical settings due to nonspecific toxicity. Thus, a major advantage of DELVR is that EEPs are hidden by the dense DNA shell and can be selectively exposed to a single molecule with duplex denaturation. We show that EEPs in our platform boost antisense efficacy without detrimental effects on the i-Motif trigger, which enables its application for SNA delivery. As SNAs are reported to remain trapped within endosomes and are degraded by lysosomes, DELVR solves a major limitation for SNAs. We chose HIF1a as our antisense target as it is a prevalent target for cancer therapies that is upregulated alongside the hypoxic conditions often associated with tumors.

We demonstrate the ability of the DELVR platform to enhance the efficacy of established antisense drugs by using EZN2968 to target HIF1a, a prevalent target for cancer therapies. An added benefit of this platform is the enhanced tumor localization resulting from the use of an SNA delivery agent. The modular design allows for the antisense drug, EEP, and even the nanoparticle vehicle to be exchanged, depending on application. By testing various DELVR components individually, we show that the highest level of antisense activity is found when all of the components are combined. This activity even rivals nonclinical solutions such as cationic transfection agents, further demonstrating the platform’s capability. To further understand the factors that govern DELVR’s high activity levels, we show that the endosomal escape efficiency is diminished when the i-Motif duplex anchor is exchanged with a non-pH-responsive duplex, which highlights that the activity is primarily due to the pH-triggering mechanism rather than nonspecific or nuclease dissociation. Additionally, we demonstrate the potential of FLIM techniques, as we found that the synergistic DELVR construct had the fastest release within HeLa cells across all four DELVR variants. This suggests that rapid cytosolic entry enhances the antisense therapeutic and may reduce nonspecific lysosomal degradation as indicated by knockdown results. For many nucleic acid therapeutic strategies, delivery efficiency remains a major bottleneck toward success in the clinic, and we envision that DELVR may allow for increased clinical translation for nucleic acid therapies.

## Methods

### Materials

All chemicals purchased were used without further purification unless otherwise noted. Sodium bicarbonate (cat. no. S6014-500g), sodium citrate tribasic dihydrate (cat. no. S4641-500G), sodium ascorbate (cat. no. A4034-100G), sodium acetate (cat. no. S2889-250G), MES (cat. no. M8250-100G), hydrochloric acid (cat. no. HX0603-3), DMSO (cat. no. MX1457-7), acetonitrile (cat. no. 34998-4L), potassium cyanide (cat. no. 60178-25G), and sodium chloride (cat. no. SX0420-1) were purchased from Sigma-Aldrich. Azidoacetic acid NHS ester (catalog no. BP-22467) was purchased from BroadPharma. Tetrachloroauric(III) acid trihydrate (catalog no. 411070010) was purchased from Acros Organics. HEPES (catalog no. 5380) was purchased from OmniPur. THPTA (cat. no. F4050) was purchased from Lumiprobe. Quant-IT Oligreen ssDNA reagent (cat. no. 22360) and oligofectamine (cat. no. 58303) were purchased from Invitrogen. Opti-MEM I reduced serum medium (cat. no. 31985070), Dulbecco’s modified Eagle medium 1× (cat. no. 11995-065), and 0.4% Trypan Blue (cat. no. 15250-061) were purchased from Gibco. High-capacity cDNA reverse transcription kit (catalog no. 4368814) was purchased from Applied Biosystems. Nitric acid (cat. no. T003090500) and triethylamine (cat. no. 04885-1) were purchased from ThermoFisher. Primary antibodies (cat. no. 14-1079-80, MA5-14794), secondary antibodies (cat. no. A32733TR, A32766TR), and NucBlue Fixed Cell ReadyProbes (cat. no. R37606) were purchased from ThermoFisher. Trypsin (0.25%), 2.21 mM EDTA, 1× sodium bicarbonate (cat. no. 25-053-Cl), and fetal bovine serum (cat. no. 35-010-CV) were purchased from Corning. Cy3B-NHS ester (catalog no. PA63101) was purchased from GE Healthcare Life Sciences. Cupric sulfate and 5-hydrate (catalog no. 4844-04) were purchased from Mallinckrodt Pharmaceuticals. Triethylammonium acetate (cat. no. 60-4110-62) was purchased from Glen Research. QIAzol lysis reagent (cat. no. 79306) and RNeasy mini kit (cat. no. 74106) were purchased from QIAGEN. DNase I Set (catalog no. E1010) was purchased from Zymo Research. Penicillin/streptomycin (catalog no. K952-100 mL) and PerfeCTa SYBR Green FastMix ROX (catalog no. 95073-012) were purchased from VWR. All peptides were purchased from GenScript Biotech Corporation, stored at −30 °C, and used without purification. All oligonucleotides were purchased from Integrated DNA Technologies, stored at −30 °C, and used without further purification. Oligonucleotides were modeled using NUPACK software and Integrated DNA Technologies, OligoAnalyzer, tool. Nanopure water (Barnstead Nanopure system, resistivity = 18.2 MΩ) was used to prepare stock solutions. UB4 buffer was prepared by adding 20 mM sodium acetate, 20 mM MES, 20 mM HEPES, and 117 mM NaCl.

#### Synthesis of 15 nm Gold Nanoparticles

We prepared a 250 mL two-neck round-bottom boiling flask with a stir bar by adding aqua regia (3:1 HCl to HNO_3_) and mixing within the flask for 1 min. **Note: Aqua regia is corrosive, and care must be taken to ensure safety when handling and disposing of the solution.** Aqua regia was discarded, and the flask was rinsed at least 15 times with nanopure water. The flask was inverted and left to dry until use. A 4 mL volume of 25 mM HAuCl_4_ stock was diluted in 96 mL of H_2_O and was added to the two-neck round-bottom flask. A condenser was attached to one neck, and the other neck was covered with foil. The flask was placed over a water bath, stirred vigorously at 400 rpm, and boiled using a hot plate. Once boiling, 10 mL of 38.8 mM sodium citrate tribasic was swiftly injected into the flask and refluxed for 15 min. The flask was removed and quickly placed onto ice until cooled. The solution was concentrated by removing the supernatant via centrifugation at 13,000*g* for 30 min and stored at 4 °C until further use. The concentration was determined through UV–vis spectroscopy by measuring the peak absorbance (∼520 nm) and using Beer–Lambert’s law: = ε × *c* × l, where ε = 3.1 × 10 cm^–1^ M^–1^, *l* = 0.1 cm. Note: the extinction coefficient is dependent on the AuNP size, as determined by TEM.

#### DNA Functionalization of Gold Nanoparticles

Gold nanoparticles were functionalized with DNA following the freeze method.^65^ Thiolated DNA and its complement were added in 300-fold excess to 15 nm gold nanoparticles and frozen at −30 °C for at least 1 h. Immediately after freezer removal, 10× PBS was added to create a final concentration of 1× PBS and was thawed for 30 min. Following the thaw, the solution was brought up to 500 μL using 1× PBS before being centrifuged at 13,000*g* for 20 min at RT (Eppendorf Centrifuge 5424 R). Unbound DNA was removed via aspiration, and the gold nanoparticle solution was washed and centrifuged a total of three times. DNA-functionalized gold nanoparticles (spherical nucleic acids) were stored at 4 °C for up to 1 week until use. Before experimentation, the concentration was determined through UV–vis spectroscopy by measuring the peak absorbance (∼527 nm) and using Beer–Lambert’s law.

#### TEM Gold Nanoparticle Characterization

TEM sample grids were prepared by plasmon etching a 200-mesh copper grid for 1 min. A drop (∼5 μL) of citrate-capped gold nanoparticles was placed on the grid for 30 s before being gently wiped and dried for 2 min. Images were acquired on a Hitachi HT7700 transmission electron microscope at an 80 kV accelerating voltage. TEM images were analyzed using the “Analyze Particles” package on ImageJ software.

#### Synthesis of Dye-Functionalized DNA

Amine-modified DNA was functionalized to NHS ester modified dye (Cy3B, ATTO532, ATTO647N) via NHS ester amine chemistry. A 50 μg aliquot of NHS ester dye was suspended in 1 μL of fresh DMSO. A 1 μL volume of 10× PBS, 1 μL of 1 M NaHCO_3_, and 7 μL of 1 mM DNA were combined. Then, dye was added to the DNA solution and left to react for 1 h. The reaction was quenched with the addition of 1× TBS and run through a P-2 gel (BioRad) to remove excess unreacted dye. Product was purified through reverse-phase HPLC with an Agilent AdvanceBio Oligonucleotide C18 column and eluted in solvents A (0.1 M TEAA in H_2_O) and B (acetonitrile (ACN)). Product was eluted with a linear gradient of 10–27.5% solvent B over 35 min at 60 °C. Product was concentrated using a VacuFuge and confirmed using electron spray ionization mass spectrometry.

#### Synthesis of Azide-Functionalized DNA

Briefly, amine-modified DNA was functionalized with an azide group via NHS ester amine chemistry. This procedure was conducted for both phosphodiester and phosphorothioate DNA. A 2 μL volume of 1 M NaHCO_3_ was added to 2 μL of 10× PBS. A 10 μL portion of 1 mM amine-modified DNA was added to the solution. An excess of azidoacetic acid NHS ester (1 mg) was prepared in 25 μL of DMSO and was added to begin the reaction. The reaction was left for 1 h at room temperature and quenched with addition of 1× TBS. The product was purified using reverse-phase HPLC with an Agilent AdvanceBio Oligonucleotide C18 column and eluted in solvents A (0.1 M TEAA in H_2_O) and B (ACN). Product was eluted with a linear gradient of 10–27.5% solvent B over 35 min at 60 °C. Recovered product was concentrated using a VacuFuge (Eppendorf) and confirmed via electron spray ionization mass spectrometry (Thermo Scientific LTQ Orbitrap Velos) as described below.

#### Copper Click Cycloaddition of Endosomal Escape Peptides to DNA

Azide-modified DNA was functionalized to endosomal escape peptides through copper click cycloaddition. A 10 μL amount of 20 mM CuSO_4_, 30 μL of 50 mM THPTA, and 10 μL of TEA were combined and left at room temperature for 5 min. In a separate tube, 100 μg of EEP was dissolved in 38.5 μL of DMSO and was added to 10 μL of 1 mM DNA. After a brief incubation, 2.5 μL of 100 mM sodium ascorbate was added to the CuSO_4_–THPTA solution. Both tubes were combined and incubated at 50 °C for 1 h. To quench the reaction, 50 μL of EDTA was added and the product was purified using reverse-phase HPLC with an Agilent AdvanceBio Oligonucleotide C18 column and eluted in solvents A (0.1 M TEAA in H_2_O), B (ACN), and C (50 mM EDTA, 10% MeOH). For phosphodiester backbone DNA, the product was eluted using a linear gradient of 10–40% solvent B over 30 min at RT. For phosphorothioate-modified DNA, the column was equilibrated with 100% solvent C, and the product was eluted in 100% solvent C for 12.5 min before transitioning to a linear gradient of 90% solvent A with 10–35% solvent B over 30 min at RT. Recovered product was concentrated using a VacuFuge and confirmed via electron spray ionization mass spectrometry (Thermo Scientific LTQ Orbitrap Velos) as described below.

#### Quantification of Spherical Nucleic Acid DNA Density

Spherical nucleic acids were prepared as above, either with Cy3-labeled complement or without. To prepare a five-point standard curve, concentrations of 2, 20, 50, 100, and 200 nM for the DNA anchor complement or i-Motif anchor were prepared in 100 μL of 1× TE buffer. The SNA samples were prepared at 0.5 nM AuNP in 100 μL of 1× TE as well. A 1 μL volume of 1 M KCN stock was added to each sample and incubated for 30 min at RT to etch the AuNP and release DNA in solution. **Note: KCN in aqueous solution must be handled in a fume hood, and precautions must be taken with buffer conditions to maintain safety.** For measuring i-Motif density without complement, 100 μL of 1× OliGreen ssDNA reagent was added to each sample and then immediately fluorescently measured via spectrophotometry (BioTek Synergy H1 hybrid multi-mode reader) using bandpass excitation and emission filters at 485/20 nm and 528/20 nm, respectively. For measuring Cy3-labeled complement DNA, sample was measured immediately after KCN incubation without OliGreen addition and was measured using bandpass excitation and emission filters at 540/25 and 590/20 nm, respectively. Concentration was determined through a linear regression and divided by the initial AuNP concentration to determine # DNA/AuNP.

#### Absorbance Characterization of i-Motif Folding

A 5 μM concentration of i-Motif DNA was prepared in 10 μL of 1× UB4 buffer at pH values from pH 5.0 to pH 8.0 in intervals of 0.25 pH. The solution was heated to 95 °C for 2 min and slowly cooled to 4 °C at −5 °C/min. The solution was left overnight at 4 °C. The following day, each solution was incubated at RT for 2 h and the absorbance spectrum was measured via UV–vis spectroscopy (Nanodrop 2000c) from 220 to 350 nm. The 295 nm absorbance was normalized to the isosbestic point at 280 nm absorbance to reduce concentration-related error. The p*K*_a_ was determined by fitting using a Boltzmann sigmoidal equation in GraphPad prism software.

#### Fluorescence Characterization of i-Motif Duplex Release

The duplex solution was prepared by using a 1.05:1 ratio between quencher DNA and Cy3 complement DNA in 1× PBS. The duplex was annealed at 95 °C for 3 min and slowly cooled to RT. The duplex was added to 1× UB4 buffer at pH values ranging from pH 5 to pH 8 in intervals of 0.5 pH so that the final quencher DNA and Cy3 complement DNA concentrations were 52.5 and 50 nM, respectively. Each sample was incubated at 37 °C for 3 h and was fluorescently measured using a spectrophotometer at 37 °C (BioTek Synergy H1 hybrid multi-mode reader) with bandpass excitation and emission filters at 540/25 nm and 590/20 nm, respectively.

#### Fluorescence Characterization of SNA i-Motif Release

SNAs were added at 0.5 nM concentration to 1× UB4 buffers at pH ranging from 5.0 to 8.0 in intervals of 0.5 pH. Samples were incubated for 3 h at 37 °C in a shaker at 250 rpm. After 2 h of incubation, the positive control group was heated to 65 °C for 2 min before slowly cooling to 37 °C, where it remained until the 3 h incubation had completed. Samples were fluorescently measured using a spectrophotometer at 37 °C (BioTek Synergy H1 hybrid multi-mode reader) with bandpass excitation and emission filters at 540/25 and 590/20 nm, respectively.

#### RT-qPCR to Assess HIF1a Levels after Antisense Drug Treatment for Knockdown

A total of 2.5 × 10^4^ HeLa cells were seeded in a culture-treated 24-well plate in DMEM a day before the experiment. Cell media was aspirated from the plate and washed with sterile PBS once, and 200 μL of serum-free OptiMEM was added for at least 20 min before addition of ASO drug. Briefly, ASO concentration was prepared in 5× experimental concentration in 50 μL volumes. For OFA samples, 1.5 μL of OFA reagent was diluted to a 7.5 μL volume in OptiMEM and incubated at RT for 5 min. Meanwhile, ASO was diluted to 40 μL volume in serum-free OptiMEM. After the 5 min incubation, both OFA reagent and ASO were combined and incubated for 15 min at RT. Afterward, the OFA solution was added to cells. For non-OFA samples, samples were prepared in OptiMEM with a 50 μL volume at RT and were added to wells. After a 4 h incubation, 125 μL of 30% FBS DMEM was added to each well, and cells were left to incubate for the remainder of 24 h. All samples were incubated for 24 h unless otherwise specified. After incubation, media was aspirated, and cells were lysed using 300 μL of QIAZOL lysis reagent. Total RNA was collected following the procedure described by the QIAGEN RNeasy mini extraction kit. RNA samples with poor A260/A280 ratio or <20 ng/μL concentration were discarded. RNA was reverse transcribed following the high-capacity cDNA reverse transcription kit using a thermal cycler (BioRad T100 thermal cycler). HIF1a mRNA levels were quantified following the PerfeCTa SYBR Green FastMix RT-qPCR two-step protocol with 50 μM custom primers (Supporting Table 1) in a lightcycler (Roche Lightcycler 96). Relative mRNA quantification was performed using the ΔΔC_t_ method, with 18S as an internal control.

#### Confocal Microscopy

A total of 5 × 10^3^ HeLa cells (5 × 10^3^) were plated in a black 96-well optical plate a day before the experiment. Wells were washed once with 10% FBS DMEM before 250 nM Cy3-labeled DNA was added and incubated in solution for 1 h. After incubation, wells were washed twice with HBSS and were imaged using a Nikon Ti2 Eclipse confocal microscope with a Plan Apo Lambda 60×/1.40 oil objective. Cy3 fluorescent images were captured using a C2Si laser scanning system with a 561 nm laser at 10% laser power with a 40 μm pinhole and were analyzed using Nikon Elements 4.40 and ImageJ.

#### Colocalization Analysis

A total of 5 ×10^3^ HeLa cells were plated in a Ibidi-treated eight-well slide and left overnight to adhere. The next morning, cells were incubated with 150 μL of OptiMEM for 20 min before DELVR-EEP or DELVR without EEP was added at a 5 nM final concentration in 200 μL of OptiMEM. Note that this concentration refers to the AuNP core. After 4 h, cells were washed with 10% FBS DMEM and left incubating for 4, 8, or 16 h. After incubation, cells were washed with 1× HBSS and fixed using 4% formaldehyde in 1× PBS for 15 min at room temperature. After fixation, cells were washed three times with 1× PBS before permeabilizing with 0.2% Triton-X-100 in 1× PBS for 15 min at RT. Wells were washed three times with 1× PBS-T for 5 min each at RT before blocking with 3% BSA in 1× PBS for 60 min at RT. Next, wells were washed once with 1× PBS before overnight incubation at 4 °C with anti-EEA1 and anti-LAMP1 primary antibodies in 1% BSA 1× PBS buffer. The following morning, wells were washed three times with 1× PBS-T for 5 min each at RT before secondary antibody incubation (Alexa Plus 488 and Alexa Plus 647) for 60 min. NucBlue Fixed Cell ReadyProbes reagent was also added following manufacturer guidelines. After 60 min of incubation, cells were washed three times with 1× PBS and imaged using confocal microscopy.

Briefly, images were captured using 405, 488, 561, and 640 nm lasers. Lasers were sequentially pulsed to reduce fluorescent signal bleed-through, and Z-slices were collected with the pinhole set at 1 AU. The entire Z-stack images were analyzed using ImageJ and the JaCoP plugin. Thresholds were determined using automatic Costes’ thresholding, and only slices with 100% *P* value correlation were analyzed as determined through the randomized Pearson Costes’ 2D mask method. The thresholded Manders’ coefficient (M2) was used for colocalization analysis for individual cells.

#### Fluorescence Lifetime Imaging Microscopy

A total of 5 ×10^3^ HeLa cells were plated in a black 96-well optical plate a day before the experiment. Wells were washed once with 10% FBS Fluorobrite DMEM before 2 nM DELVR constructs (∼180 nM complement DNA) were added and incubated with cells for 30 min. Wells were washed twice with 10% FBS Fluorobrite DMEM and left to incubate for various time points within 24 h. Immediately before microscopy, NucBlue live cell stain was added to stain the nuclear region following manufacturer guidelines. Imaging was performed on a Nikon Ti Eclipse inverted confocal microscope with a Plan Apo Lambda 60×/1.40 oil objective. The confocal microscope is equipped with a Picoquant laser scanning microscope TCSPC upgrade with SymPhoTime 64 software. FLIM images were collected as a 512 × 512-pixel image for five frames with a 40 μs dwell time and 40 μm pinhole using a PMA hybrid 40 dual detector. Samples were excited using pulsed interleaved excitation (PIE) with pulsed 520 ± 10 nm and 405 ± 10 nm diode lasers at 26.67 MHz. The laser light was split using a 560 nm long-pass dichroic filter into detector 1 collecting nuclear emitted photons that passed a 483/40 nm bandpass filter and detector 2 collecting ATTO532-DELVR emitted photons that passed a 582/75 nm bandpass filter. Real-time signal was attenuated to reduce the photon pile-up effect (signal <1% laser pulse rate). Data were processed using SymPhoTime64 and open source FLIMfit software^72^ with *n*-exponential reconvolution fitting algorithms for cell region pixels. An instrument response function (IRF) was measured by using a saturated and quenched erythrosine B solution in KI after imaging sessions.

#### Flow Cytometry

A total of 2.5 × 10^4^ HeLa cells were plated in a culture-treated 24-well plate a day before the experiment. Wells were aspirated, washed once with sterile PBS, and resuspended in 200 μL of OptiMEM for at least 20 min. DNA-EEP conjugates (50 nM), diluted in OptiMEM, were added to each well to incubate for 4 h. For SNA uptake experiments, 1 nM SNAs (10% ATTO647N complement and 90% complement-Aurein1.2) were incubated with cells for 1 h in OptiMEM to reduce signal from nonspecific duplex dissociation. After incubation, the wells were washed twice with sterile PBS, and cells were detached using trypsin. Cells were pelleted through centrifugation at 250*g* and resuspended in 0.5 mL of HBSS, twice. Cells were measured in a flow cytometer (Beckman Coulter Cytoflex) using a 488 nm laser with a 585/42 BP emission filter at 60 μL/min. Histograms were prepared using FlowJo software, and data were analyzed using GraphPad Prism software.

#### Cell Culture

HeLa cells obtained from ATCC were cultured in DMEM with 10% FBS, penicillin (100 U/mL), and streptomycin (100 mg/mL). OptiMEM medium was used for the transfection and uptake experiments. Cells were incubated with 100% humidity and 5% CO_2_ at 37 °C. Cells were passaged at ∼70–80% confluency following ATCC guidelines. Experiments were conducted only on cells under passage 15. Cells were counted using a hemocytometer with Trypan Blue on an Echo Rebel microscope.

## Supporting Information Available

Table of oligonucleotide and peptide sequences; additional data and experiments; further detailing of analysis techniques (PDF)
