# Supplementary material for: An Endosomal Escape Trojan Horse Platform to Improve Cytosolic Delivery of Nucleic Acids
Source: ACS Nano. 2024 Feb 12;18(8):6186–201. doi: 10.1021/acsnano.3c09027 (PMC10906071; doi:10.1021/acsnano.3c09027)
Supplement: Supplementary file 1 — nn3c09027_si_001.pdf [file nn3c09027_si_001.pdf]

An Endosomal Escape Trojan Horse Platform to Improve Cytosolic Delivery of Nucleic Acids

Steven Narum[1], Brendan Deal[2], Hiroaki Ogasawara[2], Joseph Mancuso[2], Jiahui Zhang[1], Khalid Salaita[1,2,*]

[1]Department of Biomedical Engineering, Georgia Institute of Technology and Emory University, Atlanta, GA 30322

[2]Department of Chemistry, Emory University, Atlanta, GA 30322

[*]To whom the correspondence should be addressed. Email: k.salaita@emory.edu

Supporting Table 1. Oligonucleotide sequences and structures

Name	Sequence (5' to 3')
**i-Motif 3C**	/5ThioMC6-D/TTTTTTTCGATAACGTCCCAATCCCAATCCCAATCCCT
**i-Motif 4C**	/5ThioMC6-D/TTTTTTTCGATAACGTCCCCAATCCCCAATCCCCAATCCCCT
**i-Motif 5C**	/5ThioMC6-D/TTTTTTTTTTTTTTTTCGATAACGTCCCCCAATCCCCCAAT CCCCCAATCCCCCT
**Non-pH anchor**	/5ThioMC6-D/TTTTTTTTTTTTTTTTCGATAACGTAGTAATCAATCACAGT
**PS i-Motif 5C**	/5ThioMC6-D/T*T*T*T*T*T*T*T*T*T*T*T*T*T*T*C*G*A*T*A*A*C*G* T*C*C*C*C*C*A*A*T*C*C*C*C*C*A*A*T*C*C*C*C*C*A*A*T*C*C*C*C*C* T
**PS non-pH anchor**	/5ThioMC6-D/T*T*T*T*T*T*T*T*T*T*T*T*T*T*T*C*G*A*T*A*A*C*G *T*A*G*T*A*A*T*C*A*A*T*C*A*C*A*G*T
**3CD0**	GTGATTGTGATTGTGATTGGGACGTTATCGA/3AmMO/
**3CD1**	GTGATTGTGATTGGGACGTTATCGA/3AmMO/
**3CD2**	GTGATTGGGACGTTATCGA/3AmMO/
**4CD0**	GTGGATTGTGGATTGTGGATTGGGGTCGTAATCGA/3AmMO/
**4CD1**	GTGGATTGTGGATTGGGGACGAAATCGA/3AmMO/
**4CD2**	GTGGATTGGGGACGTTTTCGA/3AmMO/
**5CD0**	GGTGGATTGGTGGATTGTGTGATTGGGGGTCGTTATTGA/3AmMO/
**5CD1**	GGTGGATTGTGTGATTGGGGGACGATATCGA/3AmMO/
**5CD2**	GTGTGATTGGGGGACGTTTTCTA/3AmMO/
**IntCy3 5CD2**	GTGTGATTGGGGGACGTTT/iCy3/TCTA/3AmMO/
**i-Motif 3C quencher**	/5IABkFQ/TTTTTTTCGATAACGTCCCAATCCCAATCCCAATCCCT
**i-Motif 4C quencher**	/5IABkFQ/TTTTTTTCGATAACGTCCCCAATCCCCAATCCCCAATCCCCT
**i-Motif 5C quencher**	/5IABkFQ/TTTTTTTCGATAACGTCCCCCAATCCCCCAATCCCCCAATCCCCCT
**Scrambled i-Motif 5C quencher**	/5IABkFQ/TTTTTTATTCCTCGCCACCACCTACTCACCACACCGCTCCTCACAC
**Scrambled 5CD2**	TTATTAGGTGGTGGCGAGTAAAA/3AmMO/
**Cy3-5CD2-AZ**	/5Cy3/GTGTGATTGGGGGACGTTTTCTA/3AZ/
**5CD2 EZN2968**	+T*+G*+G*C*A*A*G*C*A*T*C*C*+T*+G*+T*AGTGTGATTGGGGGACGTTTT CTA/3AmMO/

**PS 5CD2 EZN2968**	+T*+G*+G*C*A*A*G*C*A*T*C*C*+T*+G*+T*A*G*T*G*T*G*A*T*T*G*G*G*G*G*A*C*G*T*T*T*T*C*T*A*/3AmMO/
**PS 5CD2 EZN3088**	+C*+G*+T*C*A*G*T*A*T*G*C*G*+A*+A*+T*C*G*T*G*T*G*A*T*T*G*G*G*G*G*A*C*G*T*T*T*T*C*T*A*/3AmMO/
**EZN2968**	+T*+G*+G*C*A*A*G*C*A*T*C*C*+T*+G*+T*A/3AmMO/
**EZN3088**	+C*+G*+T*C*A*G*T*A*T*G*C*G*+A*+A*+T*C/3AmMO/
**Non-pH complement EZN2968**	+T*+G*+G*C*A*A*G*C*A*T*C*C*+T*+G*+T*A*A*T*T*G*T*G*A*T*T*G*A*T*T*A*C*T*A*C*G*T*T*A*T*C*G*/3AmMO/
**Non-pH complement EZN3088**	+C*+G*+T*C*A*G*T*A*T*G*C*G*+A*+A*+T*C*A*T*T*G*T*G*A*T*T*G*A*T*T*A*C*T*A*C*G*T*T*A*T*C*G*/3AmMO/
**HIF-1α forward primer**	TATGAGCCAGAAGAACTTTTAGGC
**HIF-1α reverse primer**	CACCTCTTTTGGCAAGCATCCTG
**18S forward primer**	AGGAATTGACGGAAGGGCACCA
**18S reverse primer**	GTGCAGCCCCGGACATCTAAG

KEY: +_ refers to locked nucleic acid, **_*** refers to phosphorothioate. Blue = i-Motif C-tracts. Red = intentional mismatch. Iowa Black FQ structure not available online. IDT modifications are shown below.

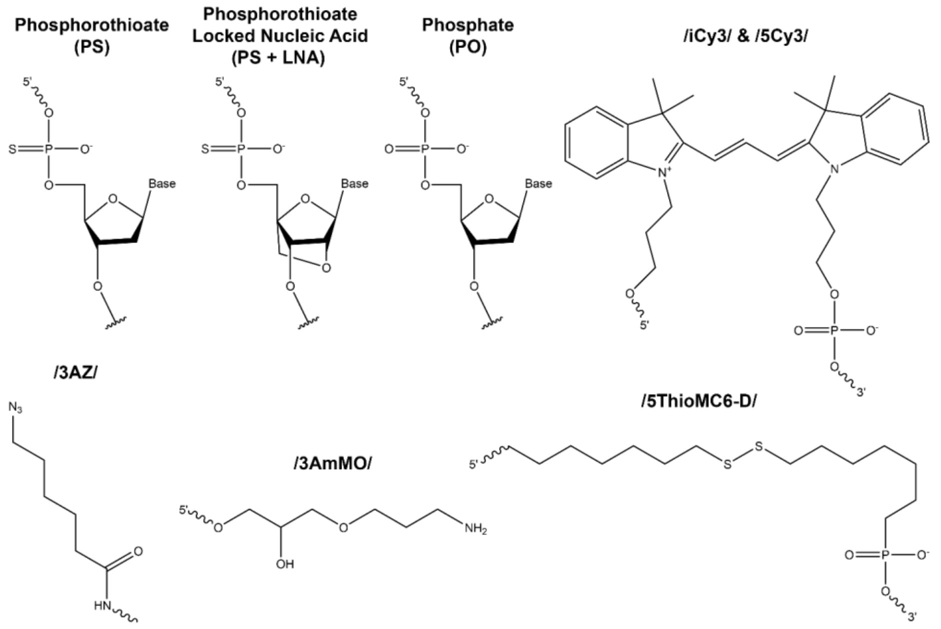

Supporting Table 2. Endosomal escape peptide sequences and structures

Name	Sequence (N to C terminus)
Aurein 1.2 N-mod	{Pra}GGGLFDIIKKIAESF
Aurein 1.2 C-mod	GLFDIIKKIAESFGG{Pra}
SP1	{Pra}GFWFG
SP2	{Pra}GWWG
SP3	{Pra}GWWWG

{Pra} refers to the noncanonical amino acid, Propargylglycine (shown below). Peptides were custom synthesized by GenScript.

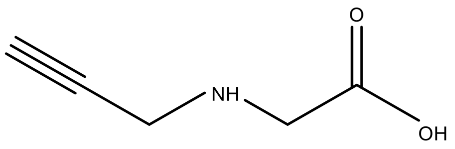

Propargylglycine

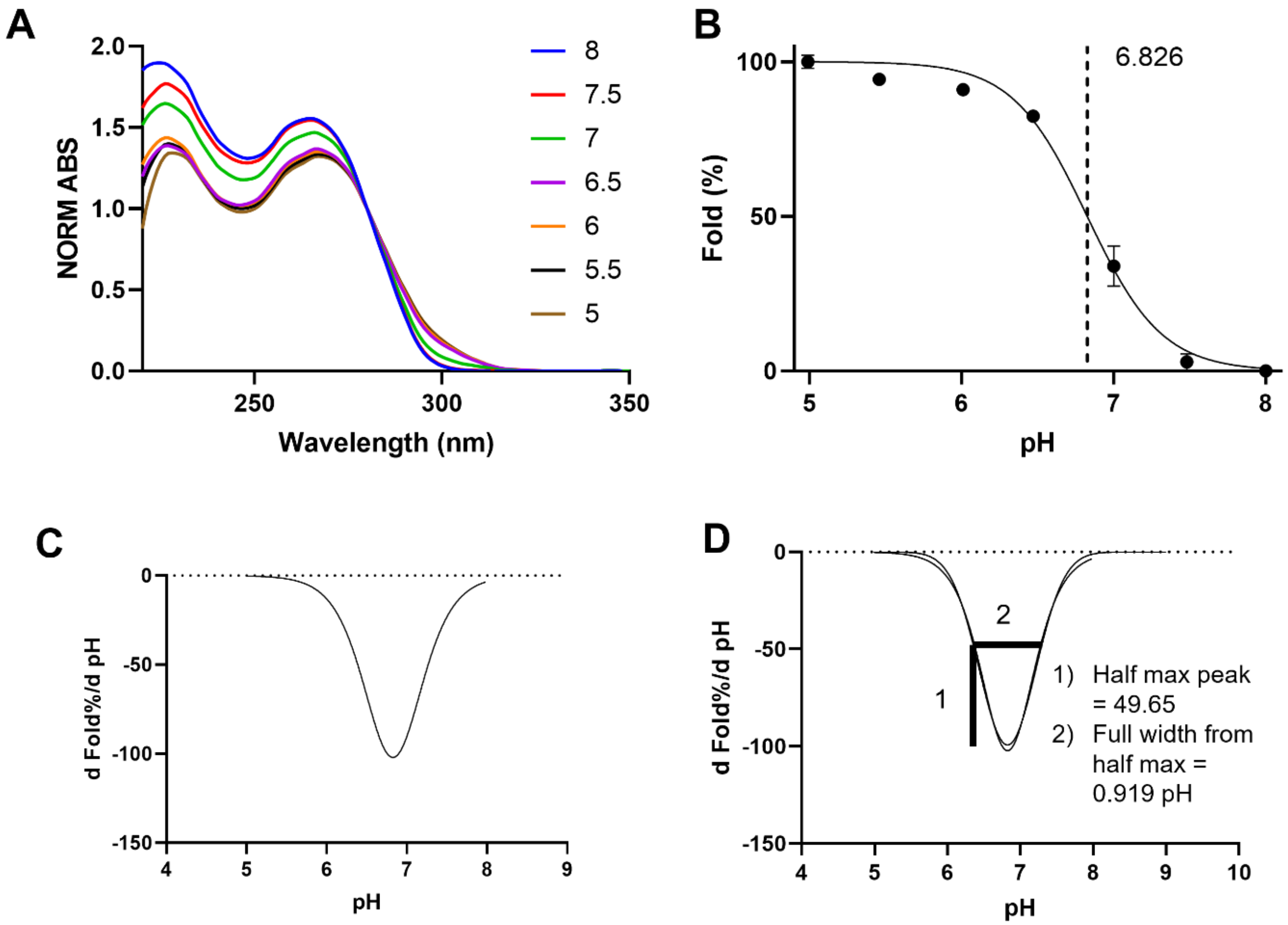

Figure S1: Single stranded PS i-Motif 5C exhibits pH-dependent structure switch. A. Absorbance spectra of PS-modified i-Motif 5C after 2h incubation at RT in 1X UB4 buffer at varying pH. Absorbance values are normalized to the isosbestic point at 280nm and are shown from 220 – 350nm with colors representing different pH values. **B.** Plot of normalized folding response to pH. The fold% is calculated by recording the absorbance at 295nm (i-Motif indicator) and with pH 5.0 and 8.0 set as the 100% and 0% values, respectively. Plot is fitted to a Boltzmann sigmoidal function with the pK_a shown on the graph. **C.** Plot of the first derivative function due to pH response (pH 5 to 8). **D.** Plot showing gaussian profile and calculation for full width from half max (FWHM) from first derivative function. FWHM = 0.919 pH. Experiments were conducted in triplicate with 5 μM DNA concentration in 1X UB4 buffer (157 mM Na^+ with 0 M Mg^{2+}) at RT with pH from 8.0 to 5.0 in 0.5 pH intervals.

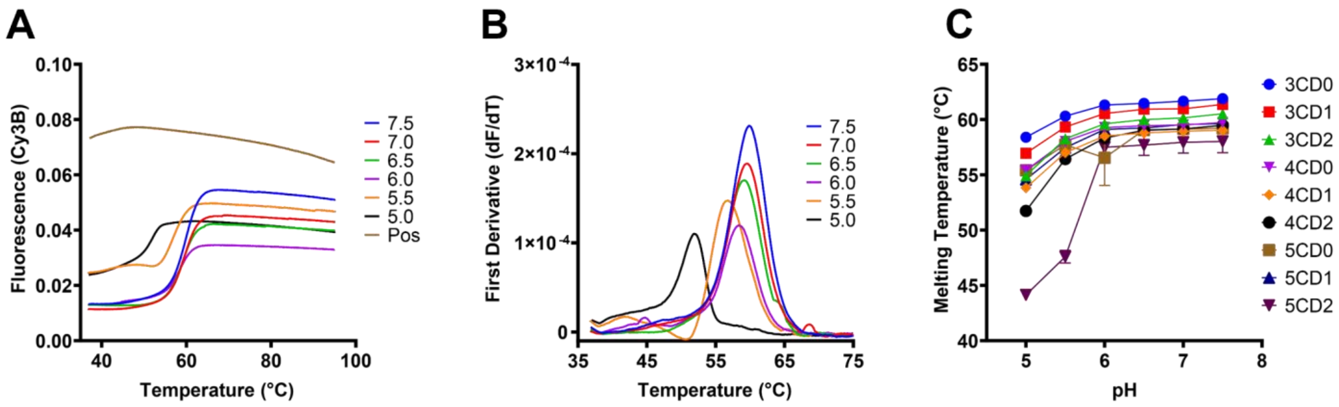

D

THEORETICAL MELTING TEMPERATURES (°C)	i-Motif 3C	i-Motif 4C	i-Motif 5C
**D0 complement**	58.2	58	58
**D1 complement**	57.1	57.3	57.4
**D2 complement**	55.2	55.5	55.5

Figure S2: Melting temperature analysis and modeling for i-Motif duplex screen. This analysis was conducted for all nine duplex pairs. **A.** Melting spectrum for the i-Motif 4C & 4CD2 complement duplex. The positive control represents a thermally denatured duplex in pH 5.0 1X UB4 buffer before melting analysis. **B.** Plot showing the first derivative of the melting spectrum used to determine melting temperature for each sample (i-Motif 4C & 4CD2 shown). The peak of each sample corresponds to the melting temperature plotted in C. The positive control is not shown as no melting spectrum was observed. **C.** Plot showing the melting temperature for all duplexes used in the folding screen at varying pH (pH 7.5 to 5). **D.** NUPACK modeling of melting temperatures for oligonucleotide pairs used in duplex screen. Experiments were conducted following methods described in the main text in triplicate with error bars representing SEM at 37 °C. Experimental conditions: 50nM Cy3B complement DNA, 52.5 nM i-Motif-Quencher DNA in 1X UB4 buffer (157 mM Na^{+} with 0 M Mg^{2+}) at 37 °C. Modeling conditions: 157 mM Na^{+}, 0 M Mg^{2+} with 100 nM for both strands.

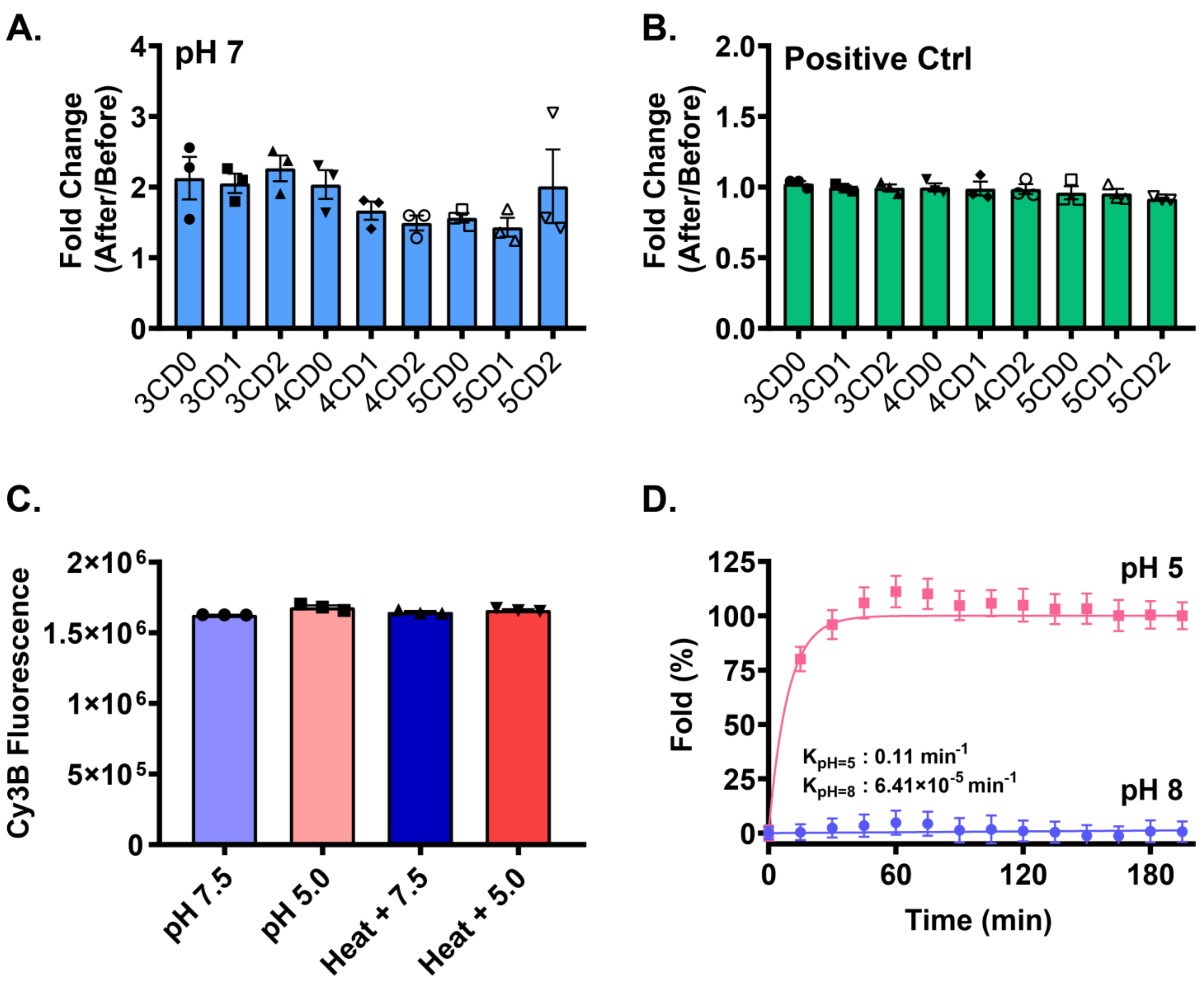

Figure S3: Characterization of i-Motif duplex stability as a function of temperature, pH, and time. A. Plot showing fold change before and after 3h incubation for each duplex at pH 7. **B.** Plot showing fold change before and after 3h incubation for each duplex with the positive control. Positive control indicates duplex that was thermally melted at 65 °C for 2min before 3h incubation at 37 °C at pH 5.0. **C.** Plot showing single stranded DNA-Cy3B is pH- and heat insensitive for 3h experiment duration. **D.** Plot showing time dependent denaturation of i-Motif duplex. Experiments were conducted in triplicate with the error bars represent SD. Experimental conditions: 50nM Cy3B complement DNA, 52.5 nM i-Motif-Quencher DNA in 1X UB4 buffer (157 mM Na^+ with 0 M Mg^{2+}) at 37 °C.

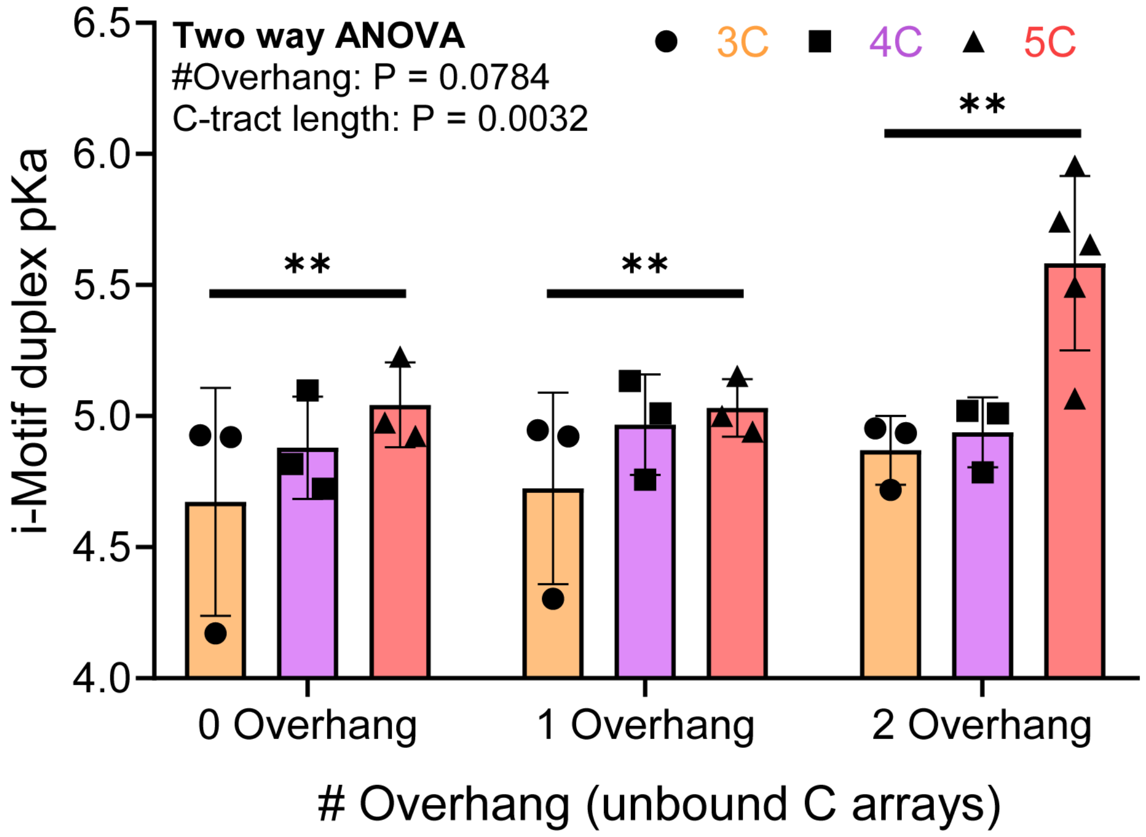

Figure S4. Two-way ANOVA analysis of i-Motif Duplex screen. Quantification of measured duplex pK_a values as a function of #overhangs and C-tract length. Nine i-Motif duplexes were screened, and each individual trial was fit using a Boltzmann sigmoidal function to determine the pK_a. The orange values represent 3C tract duplexes, the purple values represent 4C tract duplexes, and the red values represent 5C tract duplexes. The minimum and maximum were the %release values from pH 7.5 and the positive control, respectively. A two-way ANOVA was conducted with post-hoc Tukey's test for individual comparisons upon significance. Measurements were taken in at least triplicate. P values are reported as ns ($P > 0.05$), * ($P < 0.05$), ** ($P < 0.01$), *** ($P < 0.001$), and **** ($P < 0.0001$).

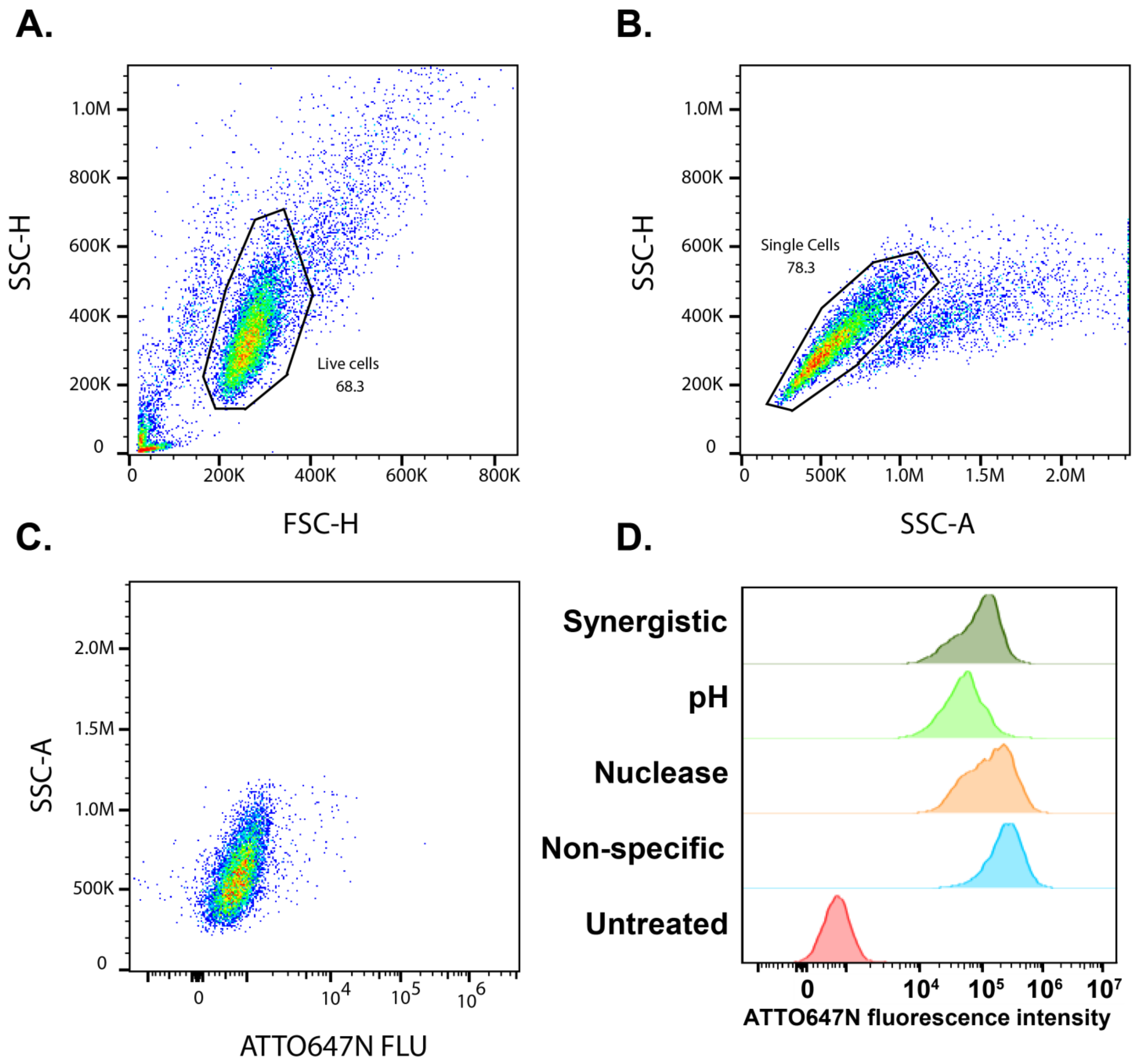

Figure S5: Gating strategy for all flow cytometry experiments. A. Cell debris is gated using forward and side scattering height profile with a gate located on the live cell population. **B.** Singlets are filtered using the side scattering height vs amplitude profile to remove noise or false positive signal in flow cytometry. **C.** Example data of post-filtered flow cytometry data that is with reduced noise to measure the mean fluorescence intensity. **D.** Example histogram data showing how cellular uptake was quantified and compared. This gating strategy was conducted for all flow cytometry experiments. Briefly, cells were treated with a fluorescent DNA sample (either 50nM Cy3-ssDNA-EEP for EEP screen or 1nM ATTO647N-dsDNA-EEP SNA for endosomal efficiency experiment) and incubated with HeLa cells for 4h (EEP screen) or 1h (endosomal efficiency) in OptiMEM media. Cells were trypsinized and detached following flow cytometry method described in the main text and resuspended in 1X HBSS for measurements. Data shown is from Figure 6D-E in the main text.

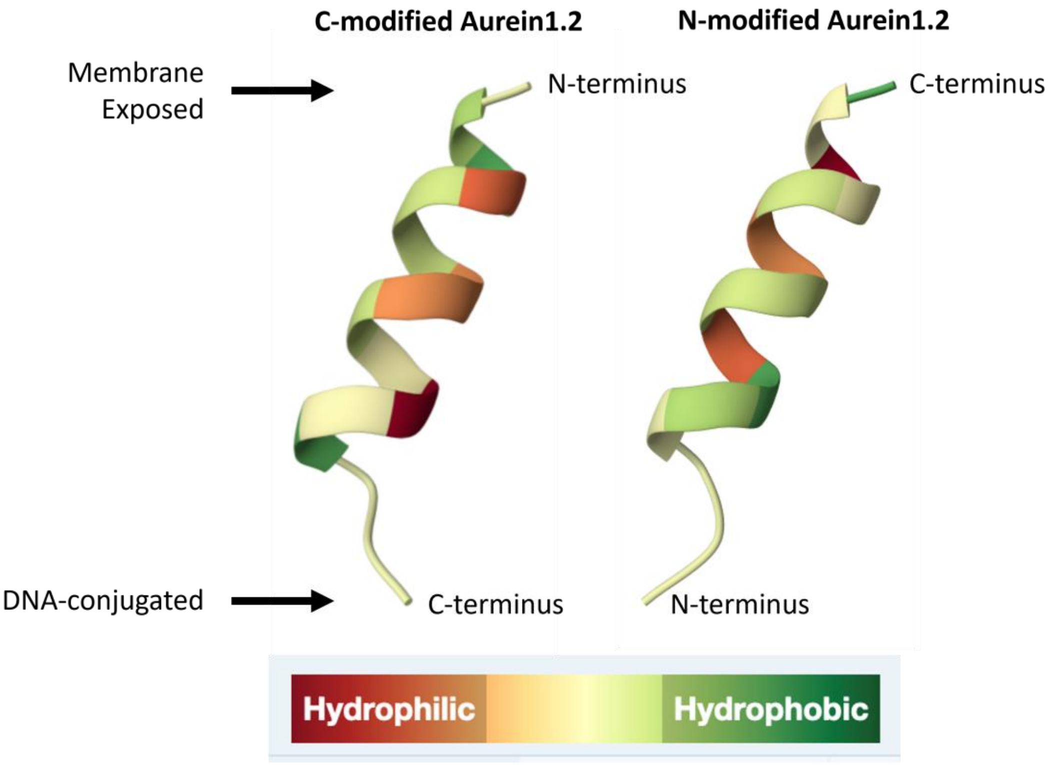

Figure S6. AlphaFold2 visualization of C-modified Aurein1.2 and N-modified Aurein1.2. A depiction the two terminal modifications and orientation of the alpha helical endosomal escape peptide (EEP) when exposed to a lipid membrane. Note that the EEPs are flipped to improve visibility. Hydrophobic residues are colored in green, and hydrophilic residues are colored in red as shown by the color scale. In both EEPs, a GGG linker containing a propargylglycine amino acid is added with the full sequences provided in Table S2.

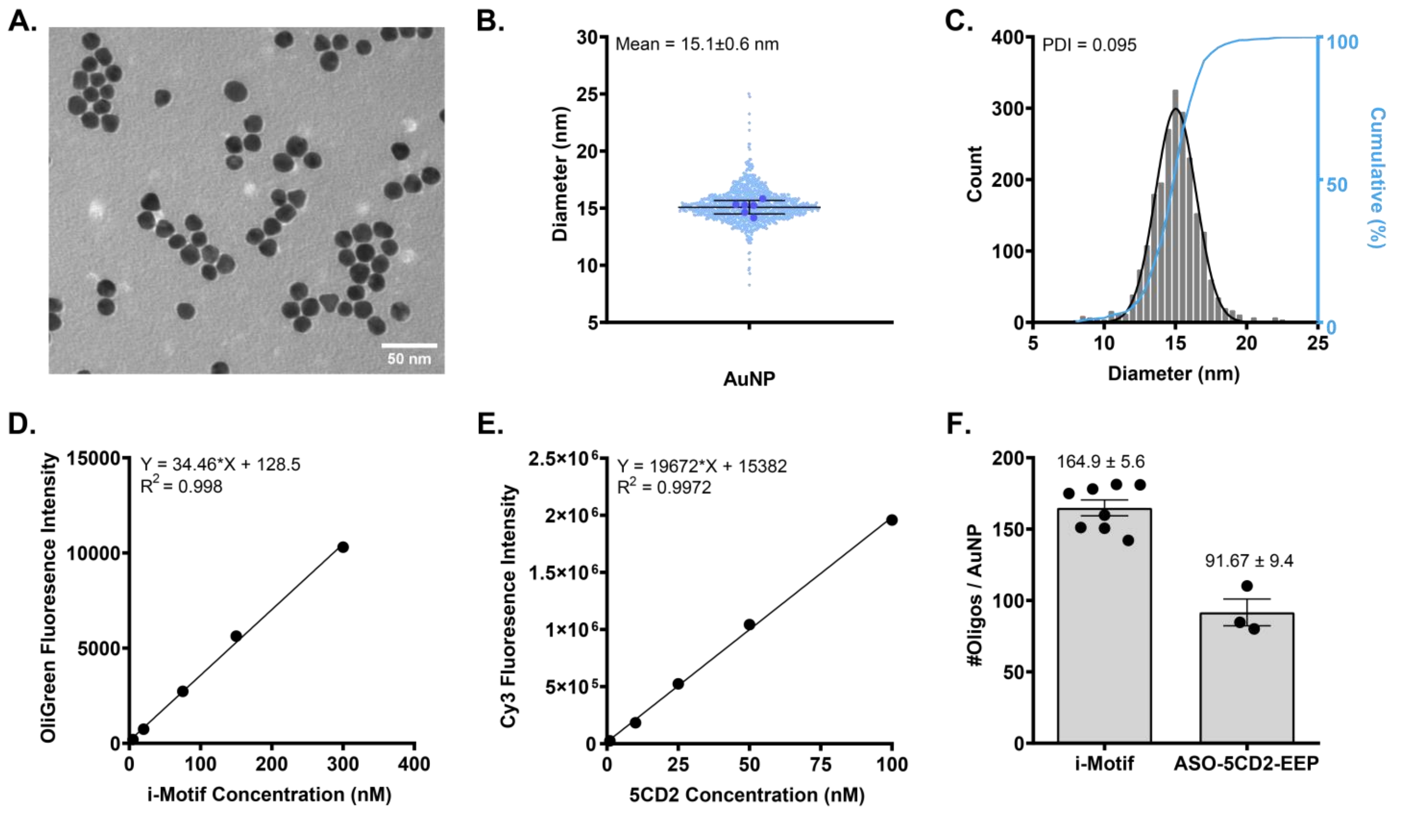

Figure S7: AuNP and SNA synthesis oligonucleotide quantification. A. Transmission Electron Microscopy (TEM) image of synthesized AuNPs. A drop of AuNPs were added to a plasmon etched 200-mesh copper grid (~30s) before being dried and imaged. Scale bar = 50nm. **B-C.** Figure showing size distribution and polydispersity index (PDI) of AuNPs determined from TEM & dynamic light scattering (DLS). Size histogram was fit using a gaussian profile to determine PDI and normality of distribution. **D.** Standard curve and linear profile for OliGreen assay to determine i-Motif anchor DNA density. DNA was diluted at varying concentrations as calculated by absorbance measurements (Nanodrop 2000c) and Beer-Lambert's Law. 100 uL of DNA was added to 100 uL of 1X TE buffer containing OliGreen assay reagent before platereader measurement (480nm excitation, 520nm emission). **E.** Standard curve and linear profile for the SNA density quantification assay to determine complement DNA density. The experimental procedure was similar to the above method with the exception that this was measured using Cy3 instead of OliGreen fluorescence. **F.** Plot showing the #oligonucleotides per AuNP for both the anchor and complement strand. SNA was prepared following freeze method (described in main text) and measured at 0.5 nM AuNP. For anchor strand quantification, SNAs were prepared without the complement strand. All experiments were conducted in at least triplicate and conducted before antisense therapy experiments to reduce batch to batch variability. Experiment was conducted at RT with varying concentrations following OliGreen assay protocol. AuNP was etched using 10 mM KCN following method described in main text.

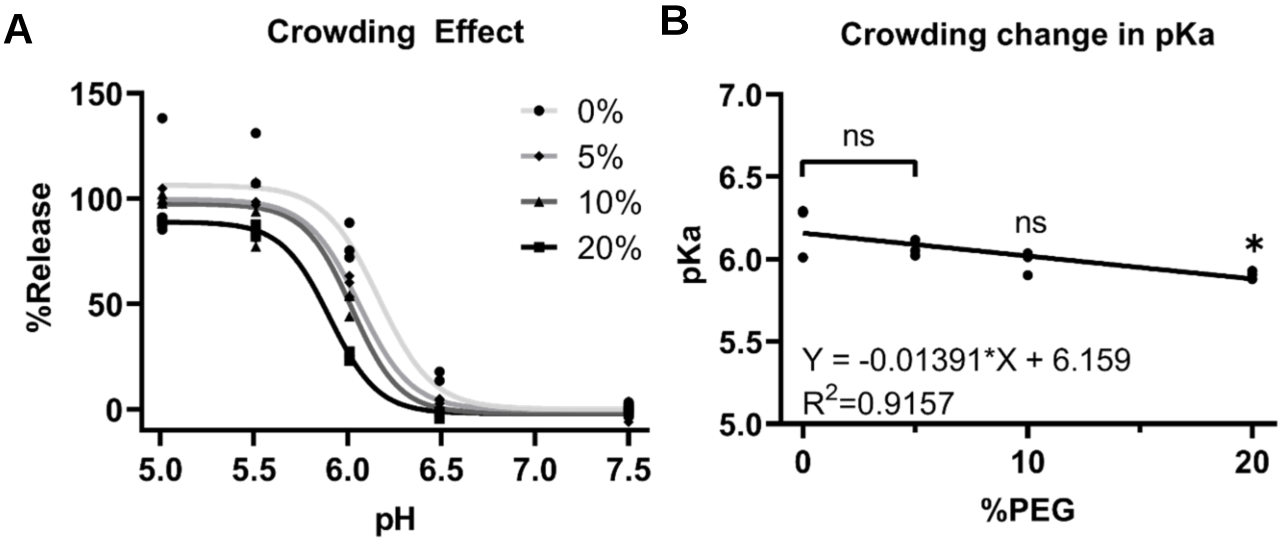

Figure S8. Molecular crowding minimally tunes pK_a of i-Motif folding. A. Effect of PEG8K in solution on i-Motif duplex release as a function of pH**.** Varying concentrations of PEG8K were prepared in 2X UB4 buffer adjusted to different pH values. The i-Motif 5C Quencher & 5CD2-Cy3 duplex were prepared by annealing at 95 °C for 3 minutes before slowly cooling to RT. The duplex was added to PEG solution with a final concentration of 1X UB4 buffer (157 mM Na^+). The final i-Motif and complement DNA concentrations in %PEG solutions were 52.5 nM and 50 nM, respectively. Each sample was incubated at 37 °C for 3 hours and fluorescently measured using a Cy3 filter cube (540ex/590em) at 37 °C. %Release was calculated by normalizing fluorescence to a thermally melted pH 5.0 duplex (100% release) and to the sample at pH 7.5 (0% release). **B.** Linear relationship between crowding effect and pK_a shift. Statistics were conducted with a one-way ANOVA and post-hoc Tukey test with P value reported as ns (P > 0.05), * (P < 0.05). All data is in triplicate.

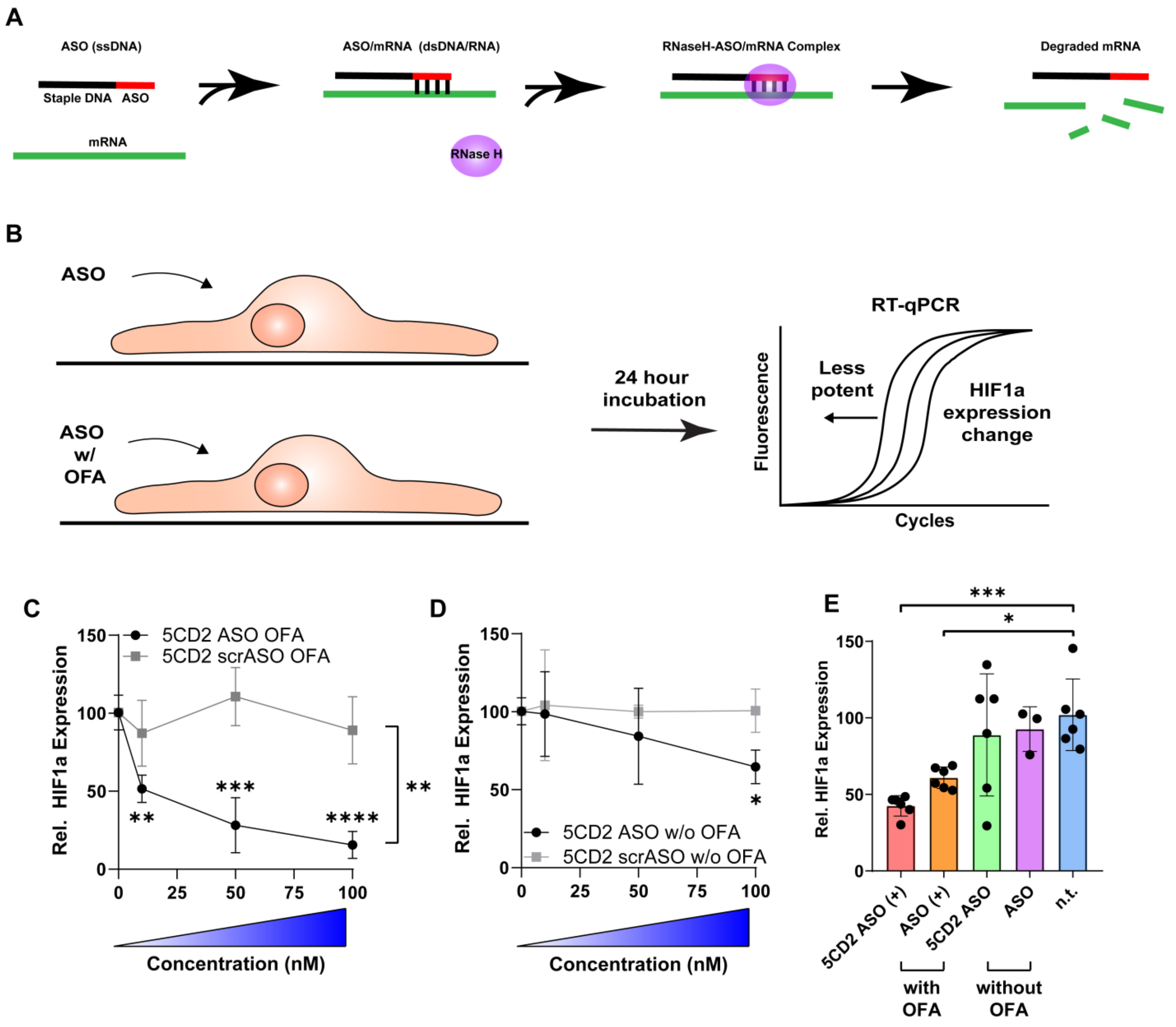

Figure S9: EZN2968 antisense drug against HIF1a is effective in HeLa cells. A. Scheme showing RNase H degradation in antisense therapy. The antisense oligonucleotide (ASO) binds mRNA, recruiting RNase H1 to degrade the target mRNA. The staple DNA does not hinder activity. **B.** Scheme showing RT-qPCR workflow and outcomes. ASO is incubated with or w/o Oligofectamine (OFA) for 24h. Purification steps not shown. Afterwards, RT-qPCR determines ASO potency through cycle time shift. **C.** Dose response for 5CD2-ASO and 5CD2-SCR in OFA at 10, 50, and 100 nM. scrASO refers to scrambled ASO sequence. Cells were incubated with DNA encapsulated in OFA for 4 hours in OptiMEM before addition of 30% FBS DMEM to create a 10% FBS final media concentration for an additional 20 hours. After incubation, cells were washed and lysed using QIAZOL and purified following protocol described in main text. RT-qPCR was conducted using HIF1a and 18S primers and normalized to the untreated control for both SCR and ASO groups. **D.** Dose response for 5CD2-ASO w/o OFA at 10, 50, 100 nM. The experiment was conducted similarly to method described above with the difference that no oligofectamine was used for ASO-5CD2 delivery. HIF1a knockdown is normalized to the respective SCR group at the corresponding DNA concentrations. **E.** Plot comparing HIF1a knockdown at 10 nM for 5CD2-ASO and ASO w/o staple. The experiment was conducted following method above with or without OFA transfection as well as with or without staple region. (+) = OFA, and n.t. is untreated expression level. HIF1a expression was normalized to a scrambled control. Statistics were performed using a one-way ANOVA with post-hoc Tukey's tests vs negative control. A t-test compared SCR vs ASO in C. SCR was not significant vs untreated control. P values are reported as ns ($P > 0.05$), * ($P < 0.05$), ** ($P < 0.01$), *** ($P < 0.001$), and **** ($P < 0.0001$). All groups were performed in triplicate.

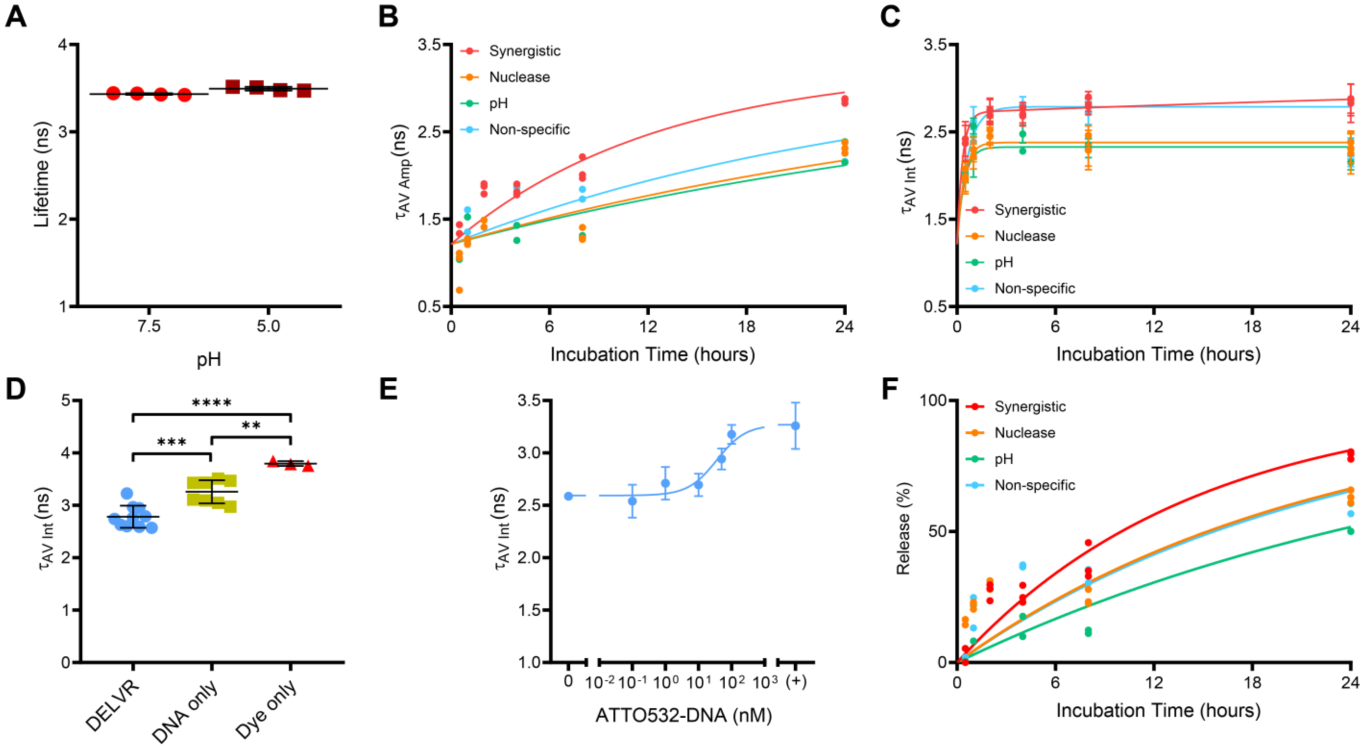

Figure S10. Fluorescence Lifetime Imaging Microscopy (FLIM) controls. A. Lifetime quantification of 100 nM ATTO532-5CD2 DNA in 1X UB4 buffer at pH 5.0 and 7.5. Note that ATTO532 lifetime is pH independent, enabling use as an endosomal reporter. **B, C.** Amplitude-weighted lifetime and intensity-weighted lifetime timelapses for all four DELVR constructs within HeLa cells. Each DELVR was incubated within HeLa cells at 2 nM concentration for 30 minutes before incubating for remainder of designated time up to 24 hours. Data was fit using a bi or mono-exponential decay depending on chi-squared and general goodness of fit. Fitted line represents a one-way association decay to evaluate cellular half-lives. **D.** Intensity-weighted lifetime quantification of an intact DELVR construct, ATTO-532 DNA, and ATTO-532 Dye. **E.** Average intensity-weighted lifetime of a titration of unbound ATTO532-DNA to 0.5 nM synergistic DELVR constructs in 1X PBS. Data is represented by increasing concentration of unbound ATTO532 DNA. **F.** Plot showing the normalized ATTO-532 DNA release over a 24-hour HeLa cell incubation. The 0% and 100% values are determined from intact DELVR and DNA only solution measurements, respectively. Error bars represent S.E.M. All experiments were conducted in at least triplicate. Biological replicates were averaged across at least 30 cells and six ROIs to minimize imaging bias. Statistical comparisons were conducted using a one-way ANOVA with post-hoc Tukey's tests for individual comparisons (bar). P values are reported as ns ($P > 0.05$), * ($P < 0.05$), ** ($P < 0.01$), *** ($P < 0.001$), and **** ($P < 0.0001$).

psSCR-5CD2-Aurein1.2

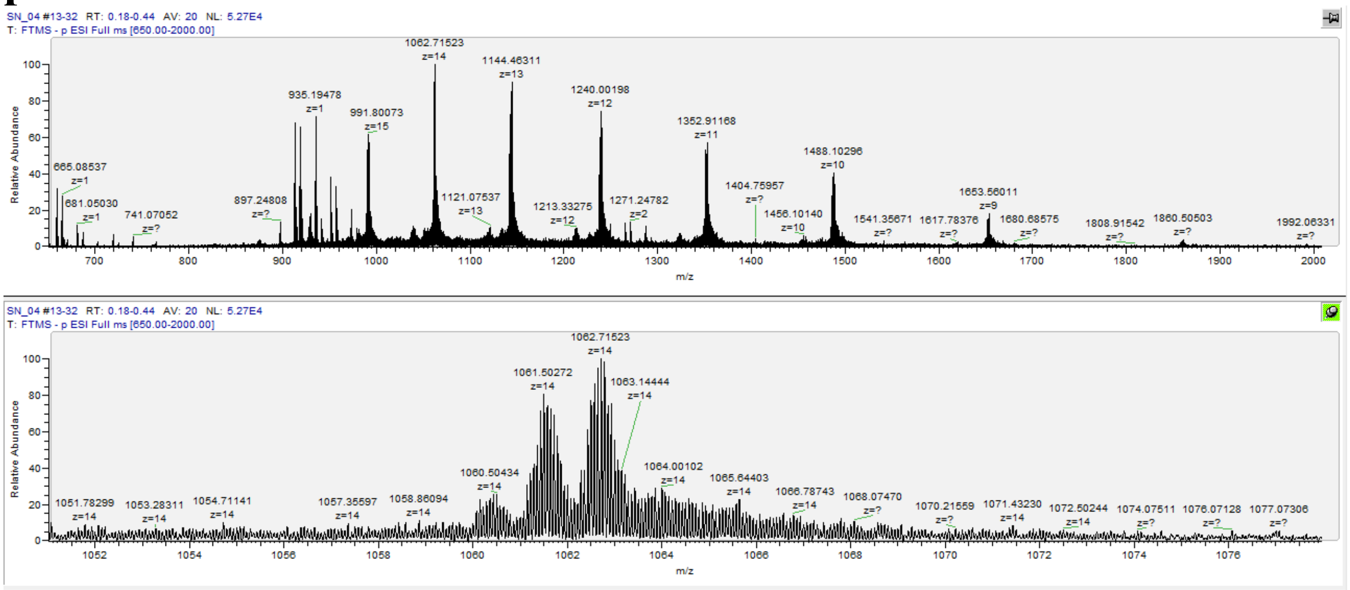

Aurein1.2

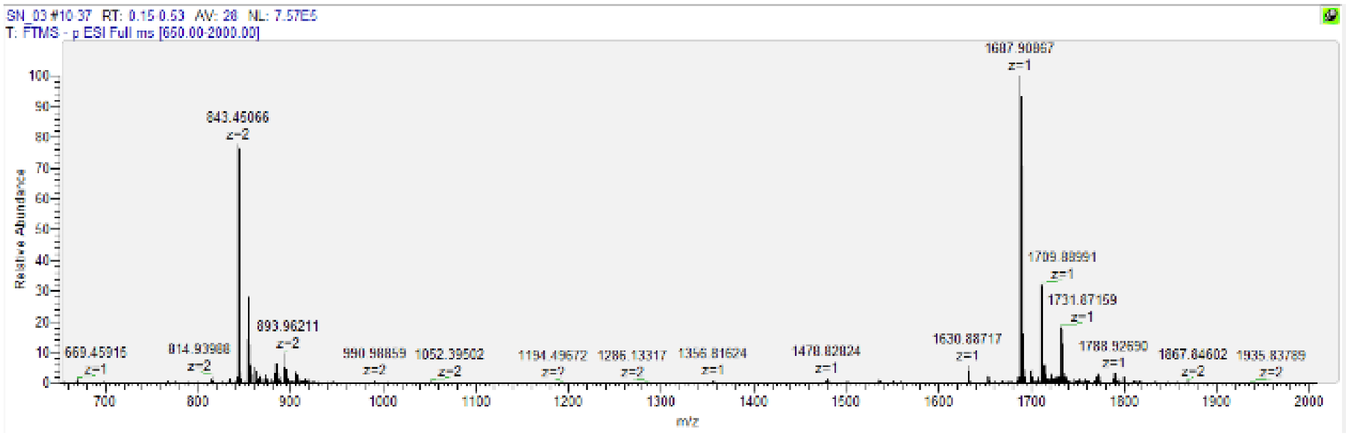

psASO-5CD2-Aurein1.2

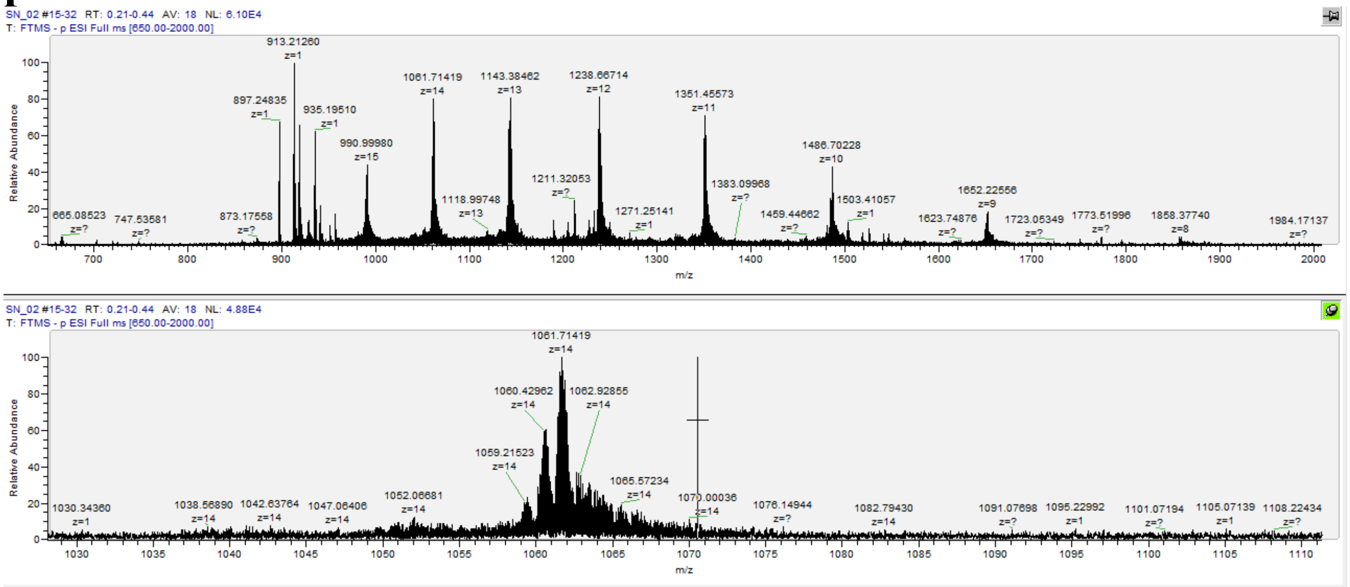

psASO-5CD2-Azide

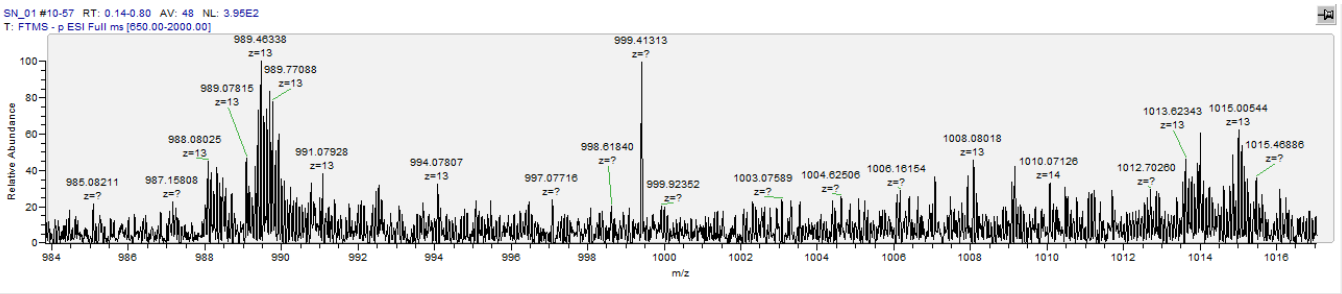

IntCy3-5CD2-SP1

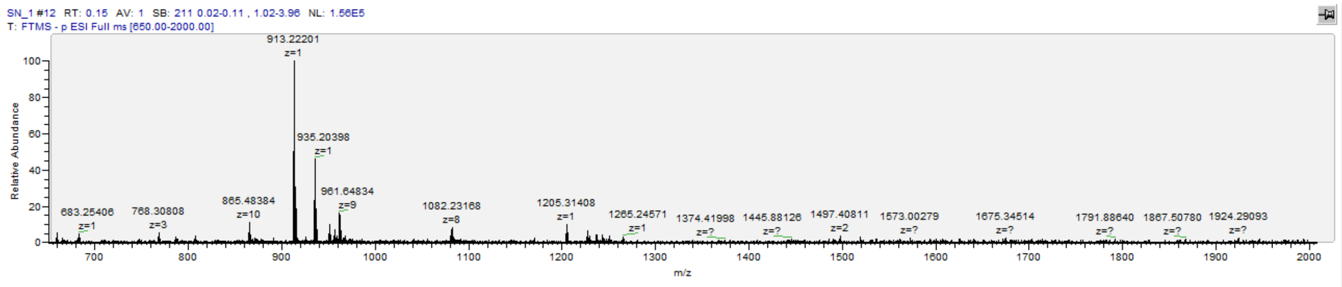

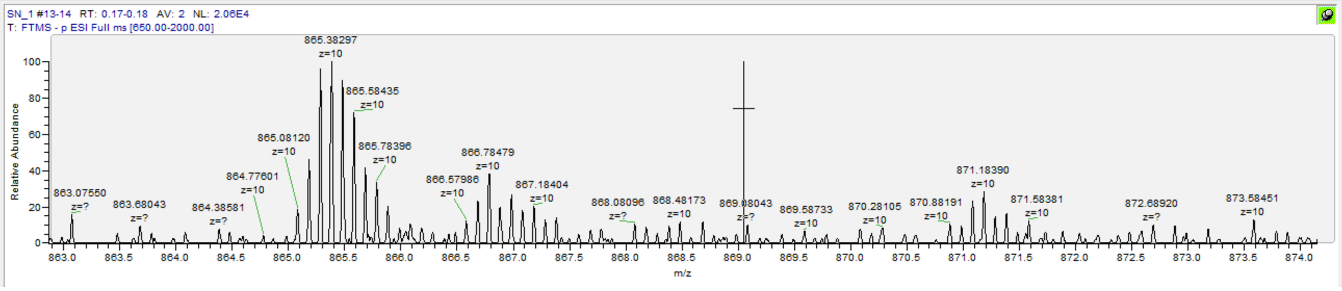

IntCy3-5CD2-Azide

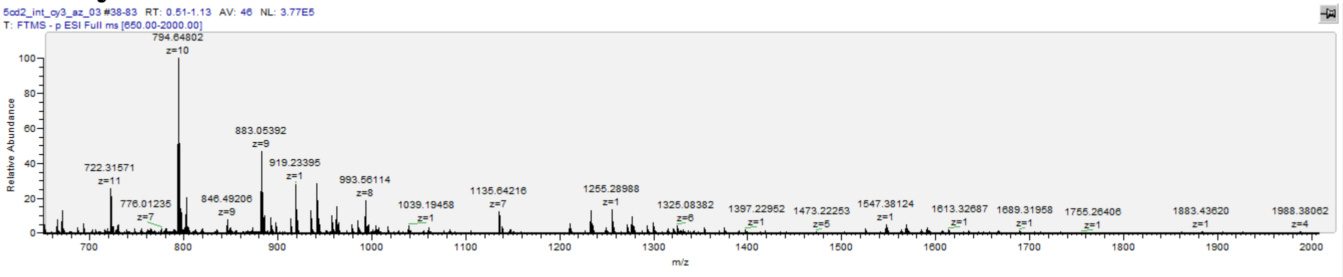

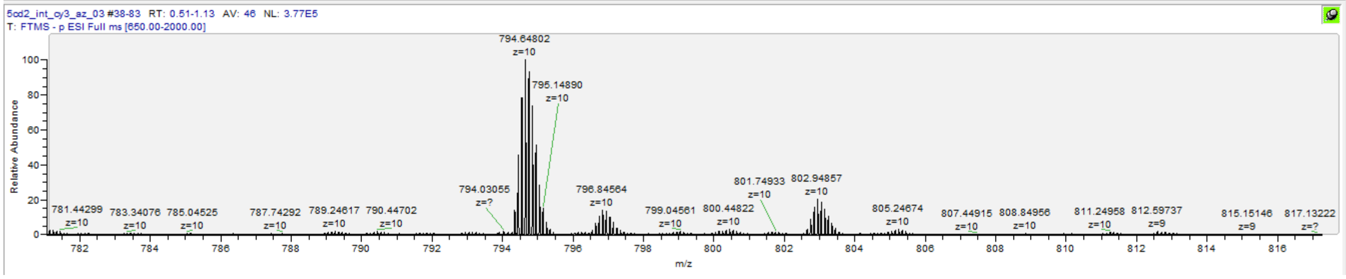

scrASO-ps5CD2-ATTO532

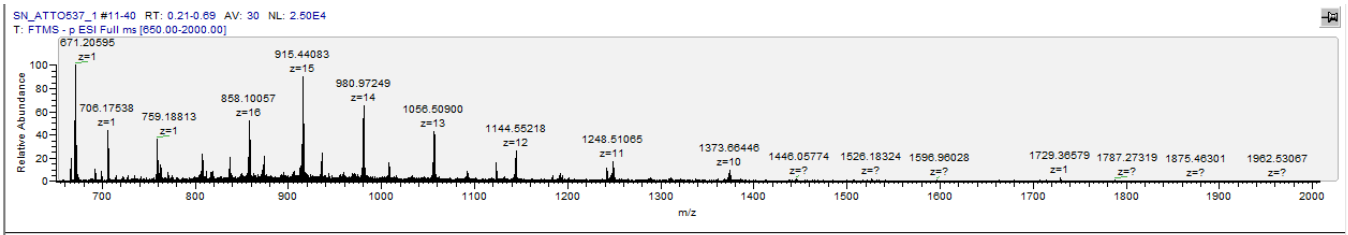

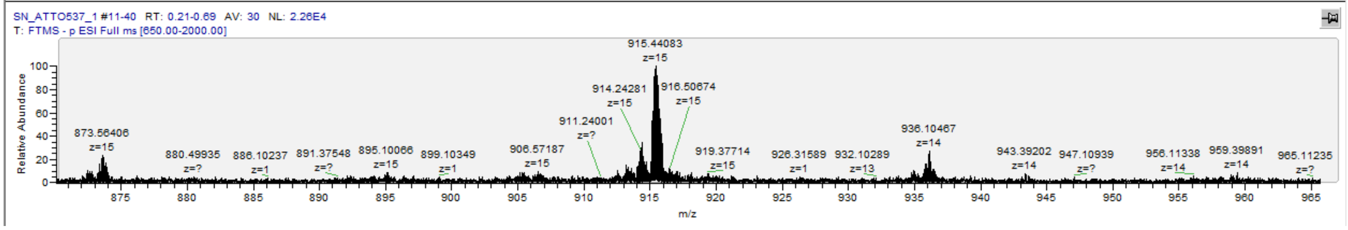

psASO-ps5CD2-CY3B

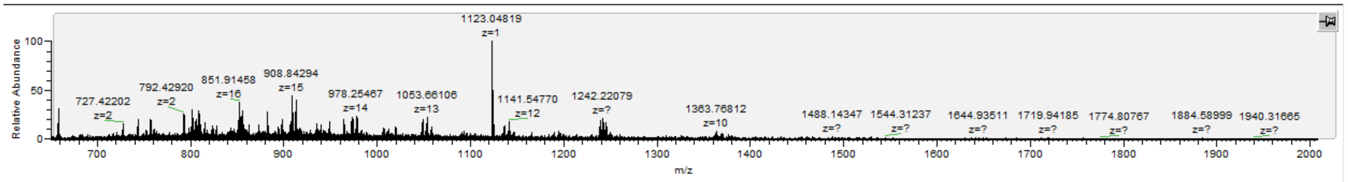

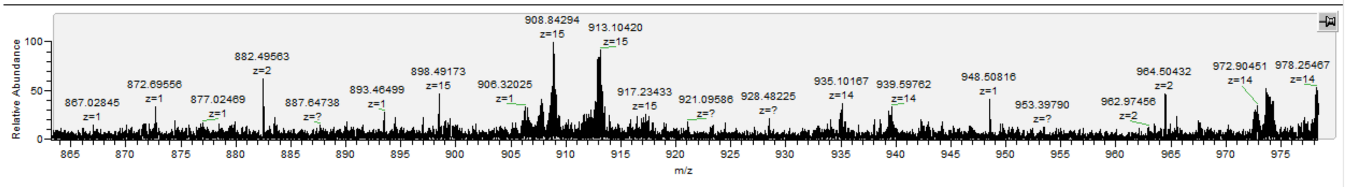

scrASO-ps5CD2-Alexa548

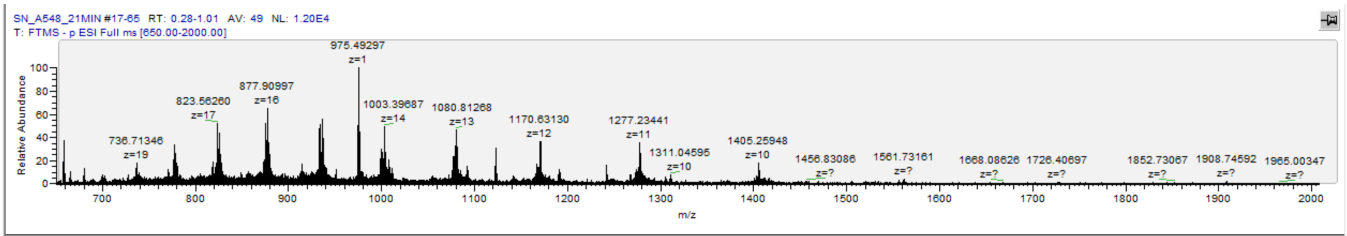

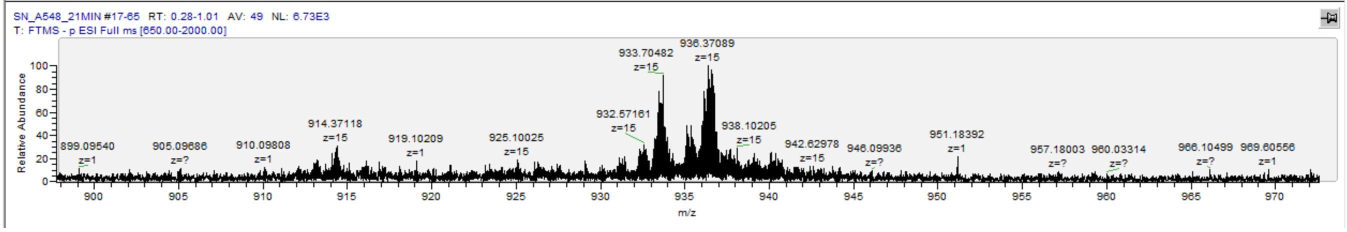

scrASO-ps5CD2-Alexa532

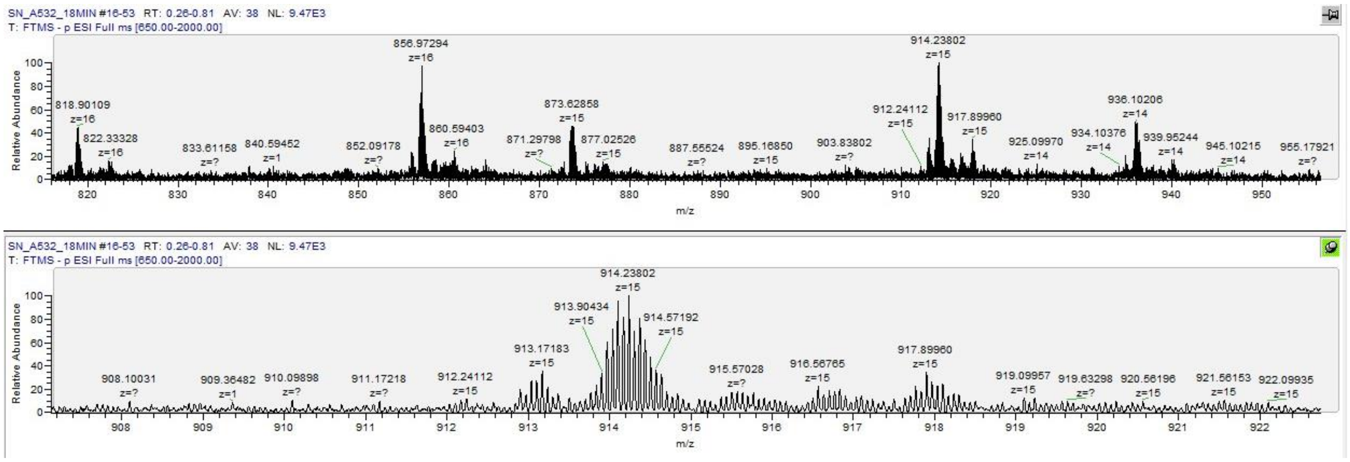

psASO-5CD2-NH2

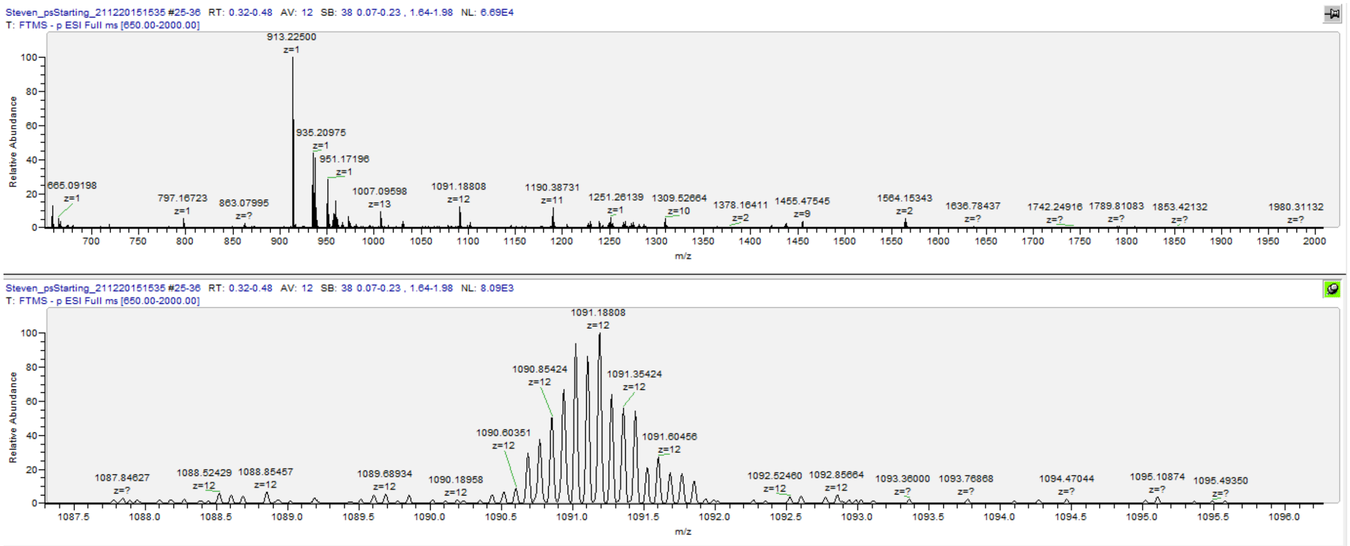

5CD2-Nmod-Aurein1.2

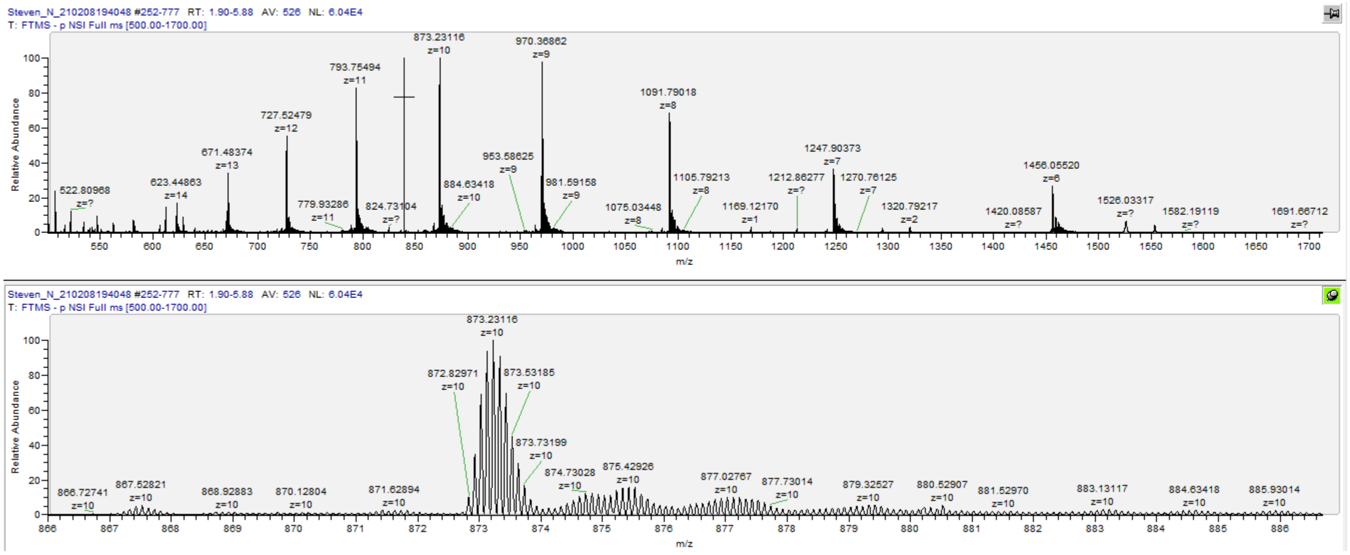

5CD2-Cmod-Aurein1.2

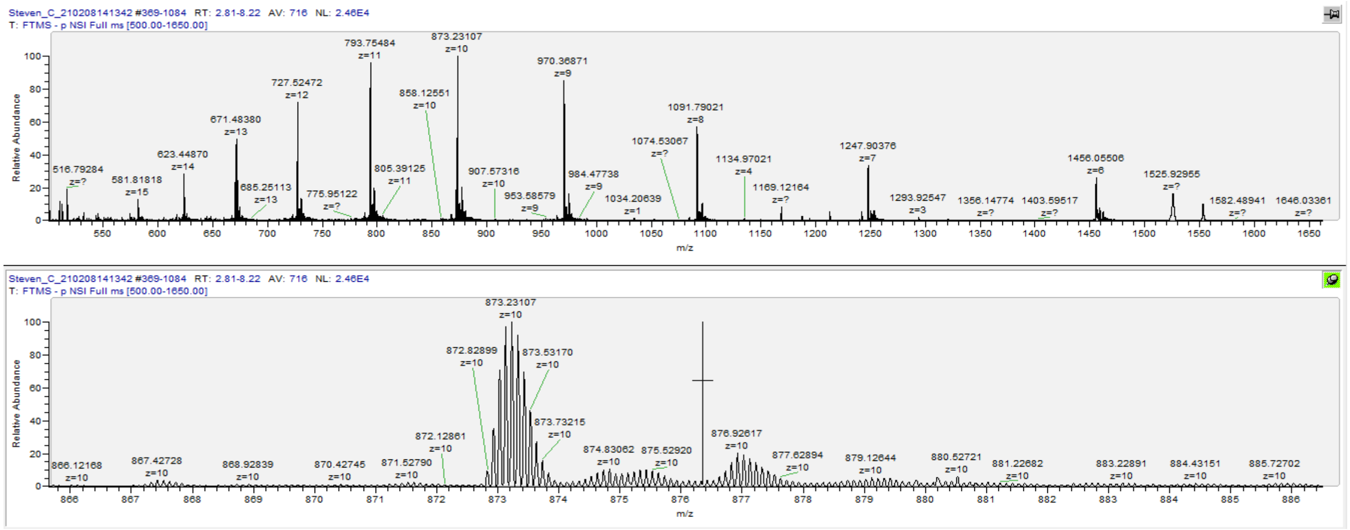

ASO-ps5CD2-SP1

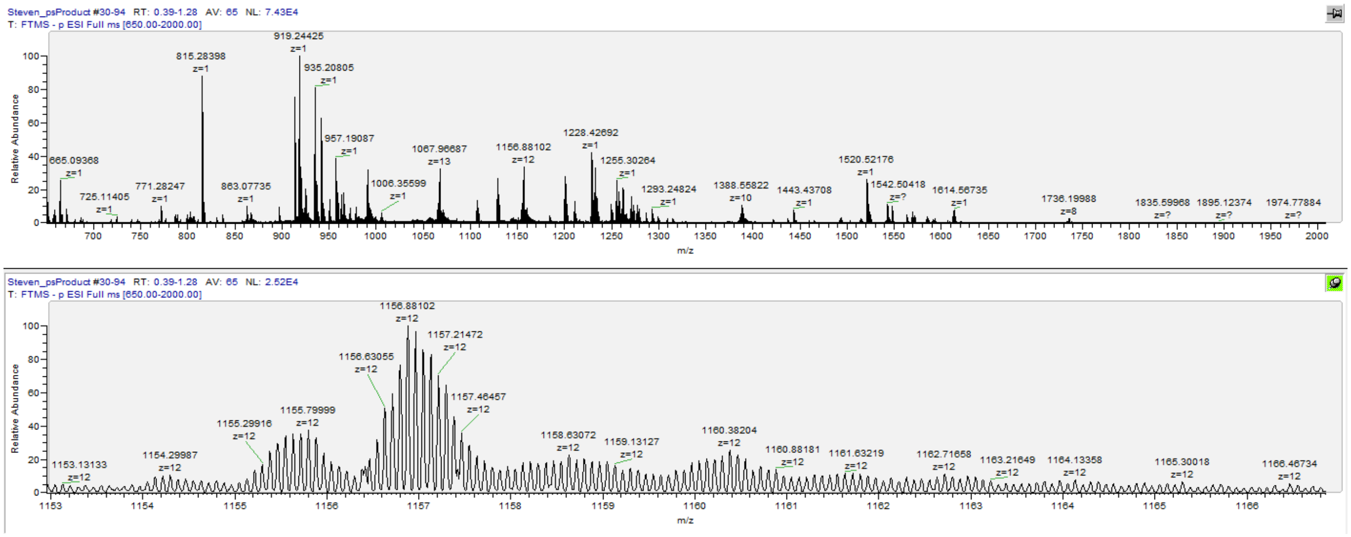

psASO-non-pH comp-Cy3B

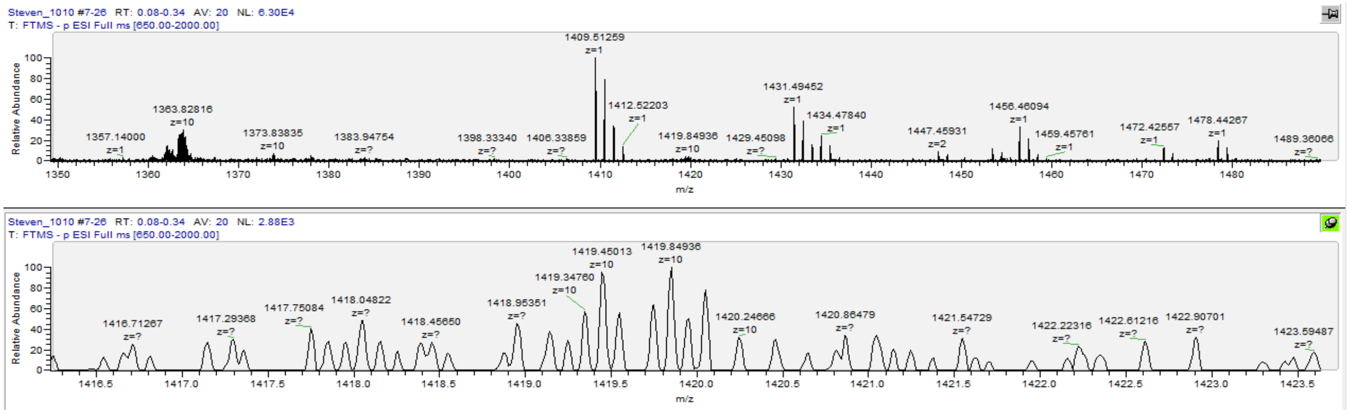

Figure S8. Electrospray Ionization Mass spectrometry (ESI-MS) characterization of DNA oligos. Oligonucleotides were reacted and purified as described in the main text. High resolution mass spectra are shown along with the respective sample name above. Supporting Table 3 shows the calculated masses and measured masses with the mass difference provided.

Supporting Table 3: Theoretical MW vs Measured ESI-MS MW for oligonucleotides and peptides

Sample	Measured MW (g/mol)	Theoretical MW (g/mol)	Difference (g/mol)
psSCR-5CD2-Aurein1.2	14891.94	14892.1	-0.16
psASO-5CD2-Aurein1.2	14877.94	14878	-0.06
Aurein1.2	1688.9	1689.6	-0.7
IntCy3-5CD2-SP1	8663.8	8664.7	-0.9
psASO-5CD2-Azide	13190.06	13188.4	1.66
IntCy3-5CD2-Azide	7957.5	7957.4	0.1
5CD2-Nmod Aurein1.2	8744.3	8741.8	2.5
5CD2-Cmod Aurein1.2	8742.3	8741.8	0.5
scrASO-ps5CD2-ATTO532	13746.6	13746.2	0.4
psASO-ps5CD2-CY3B	13647.3	13648	-0.7
scrASO-ps5CD2-Alexa532	13728.6	13728.2	0.4
scrASO-ps5CD2-Alexa548	14060.6	14061.8	-1.2
psASO-5CD2-NH2	13106.3	13105.4	0.9
psASO-5CD2-SP1	13894.6	13894.56	0.04
psASO-non-pH comp-Cy3B	14208.5	14200.5	8
